# An Alternative Approach for the Synthesis of Sulfoquinovosyldiacylglycerol

**DOI:** 10.3390/molecules26144275

**Published:** 2021-07-14

**Authors:** Tobias Sitz, Hendrik Domey, Judith Fischer, Sascha Rohn

**Affiliations:** 1 Institute of Food Chemistry, Hamburg School of Food Science, University of Hamburg, Grindelallee 117, 20146 Hamburg, Germany; tobias.sitz@chemie.uni-hamburg.de (T.S.); hendrik.domey@googlemail.com (H.D.); judith.fischer@chemie.uni-hamburg.de (J.F.); 2 Institute of Food Technology and Food Chemistry, Technische Universität Berlin, Gustav-Meyer-Allee 25, 13355 Berlin, Germany

**Keywords:** SQDG, sulfoquinovosyldiacylglycerol, sulfolipids, sulfur, total synthesis

## Abstract

Sulfoquinovosyldiacylglycerol (SQDG) is a glycolipid ubiquitously found in photosynthetically active organisms. It has attracted much attention in recent years due to its biological activities. Similarly, the increasing demand for vegan and functional foods has led to a growing interest in micronutrients such as sulfolipids and their physiological influence on human health. To study this influence, reference materials are needed for developing new analytical methods and providing enough material for model studies on the biological activity. However, the availability of these materials is limited by the difficulty to isolate and purify sulfolipids from natural sources and the unavailability of chemical standards on the market. Consequently, an alternative synthetic route for the comprehensive preparation of sulfolipids was established. Here, the synthesis of a sulfolipid with two identical saturated fatty acids is described exemplarily. The method opens possibilities for the preparation of a diverse range of interesting derivatives with different saturated and unsaturated fatty acids.

## 1. Introduction

The sulfolipid sulfoquinovosyldiacylglycerol (SQDG), first described in 1959 by Benson et al., is a polar glycolipid characterized by a sulfonic acid head group, a 6-deoxy-6-sulfo-glucose, referred to as sulfoquinovose [1]. Besides galactolipids (mono- and digalactosyldiacylglycerols), it is one of the three non-phosphorous glycolipids that form the main part of the structural lipids in the thylakoid membranes of photosynthetic organisms [2]. In addition to the anionic structures, SQDG derivatives (SQDGs) possess a high diversity of the diacylglycerol backbone due to the variation in chain length, the degree of saturation, and the fatty acid functionalization. A recent study reported up to two hundred tentatively identified sulfolipids in cyanobacteria of the genus *Arthrospira* [3]. Furthermore, SQDGs have attracted much attention in recent years because of their biological activities. Accordingly, many studies on their possible pharmacological relevance have been conducted [4,5,6]. At the same time and due to the growing demand for vegan nutrition and functional food, the interest in nutritional physiological questions regarding micronutrients like sulfolipids is increasing. However, the meaning of sulfolipids for human nutrition is still controversial. On the one hand, some health-promoting effects are attributed to them, e.g., they are known to have antiviral properties, because of their amphiphilic nature [2]. On the other hand, sulfur compounds could also have negative effects, as some microorganisms of the lower gastrointestinal tract can produce hydrogen sulfide as a (toxic) metabolite during biotransformation in the intestine [7,8,9]. Therefore, there should be the aim to improve understanding of the effects of SQDGs on human health. Consequently, it is necessary to develop new analytical methods and model systems for evaluating possible physiological effects. For such approaches, it is essential to obtain reference substances in comparatively high quantities and of appropriate purity. The difficulty here is the availability, restricted by the difficult isolation and purification of SQDGs from natural sources, and the almost non-existent commercial availability. For this reason, synthetic preparation is a crucial step. Based on this fact, it was decided to synthesize SQDGs in order to evaluate different analysis methods in the further course of research. Several approaches have been reported for the synthesis of SQDGs, but they are either quite time consuming, do not start with the simple component d-glucose, or the protecting group strategy does not provide an approach to the unsaturated lipoform. Moreover, often these synthetic strategies are not scalable due to expensive catalysts [10,11,12]. Manzo et al. (2017) developed a chemical synthesis that seemed to fulfill those criteria. However, the final deprotection of the acetyl groups required careful tuning of the experimental conditions and led to a significant decrease in yield, resulting from a partial deacylation of fatty acids by cleavage of the ester bond [13,14]. For this reason, we aimed at developing an alternative approach. It was hypothesized that a change in protecting groups after some intermediary steps of the synthesis enables a more efficient synthesis route. Further, this switch might enable preservation of the advantage of anchimeric assistance by acetate and allow an easier, gentle, and selective deprotection of the final product.

## 2. Results and Discussion

**Scheme 1:**
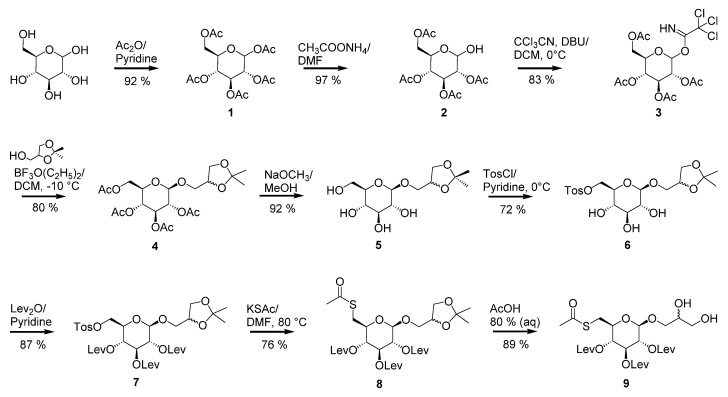
Synthesis of 3-O-[(2′,3′,4′-tri-*O*-levulinyl-6′-thioacetyl)-*β*-d-glucosyl]-(*R*/*S*)-glycerol (**9**). A precursor for potential SQDGs.

Here, an alternative and versatile strategy for the synthesis of β-sulfoquinovosyl diacylglycerol is described. The first step was to choose orthogonal protecting groups at the sugar that would allow selective deprotection without affecting the diacylglycerol moiety and the glycosidic linkage. Therefore, acetate groups were primarily chosen, which have already been used in the synthesis of α- and β-SQDGs [13,14]. Unfortunately, the use of the acetate groups in the presence of the ester linkage of glycerol to the acyl chains proved to be unsuitable. Deprotection with hydrazine monohydrate under various experimental conditions resulted in partial cleavage of the ester bond of the acyl chains at position *sn*-2. This result was also confirmed by NMR spectra, which showed shielding of the H-2′ signals and loss of the ^13^C signal for carbonyl carbons of the carboxylic acid ester. Consequently, acetate groups only as temporary protection for the hydroxy functions of the sugar moiety were used. Further in the synthesis, they were replaced by a levulinyl ester. On the one hand, acetate groups preserve the advantage of stereoselectivity in the formation of the β-glycosidic bond by anchimeric assistance [15,16], but, on the other hand, levulinic acid is more completely and selectively removable by hydrazine under mild acetate-buffered conditions, which is pushed by the formation of 6-keto-3-methyl-1,4,5,6-tetrahydro-pyridazine as a by-product [17]. As shown in Scheme 1, the synthesis begins with the acetylation of d-glucose. The reaction with pyridine as a catalyst results in an exclusive formation of the α-anomer of glucose pentaacetate [18,19]. The present result was unexpected, because a mixture of anomers, corresponding to the α/β-mixture of the anomers in the starting material, was assumed. However, the reaction conditions enable an interpretation of the results. Glucose was first dissolved in pyridine, which could have supported a base-catalyzed mutarotation to the α-anomer [20]. The subsequent addition of acetic anhydride and the reaction time of 20 h led to the formation of the thermodynamically favored α-anomer [21]. Additionally, it can be hypothesized that the recrystallization in water/methanol supports the selectivity. This first step was followed by the selective deacetylation of the anomeric hydroxyl group with ammonium acetate (compound **2**) and conversion to the trichloroacetimidate **3**. Subsequent coupling with 1,2-*O*-isopropylidene glycerol under conditions according to Schmidt et al. [22] gave the β-glycoside **4**, as diastereomeric products, determined by the neighboring group participation of the acetate group (NMR signals of the anomeric protons for both diastereomers: δ 4.59, 4.60 (d, J = 8 Hz)). It was decided to carry out the synthesis with racemic glycerol, as the primary aim was not to synthesize enantiomerically pure products, but to develop a synthesis using simple chemicals. This allows a quick and easy building up of a stock of sulfolipids for general research. Removal of all acyl protecting groups of compound **4** under Zemplen conditions gave compound **5** [23]. This was treated with tosylchloride to selectively protect the primary hydroxyl group, resulting in compound **6**. Subsequent treatment with levulinic anhydride in pyridine resulted in the fully protected product **7**. Following the replacement of the tosyl group with thioacetate gave the thioacetylglucopyranoside **8**. The next step was the removal of the isopropylidene protecting group. Treatment with 80% acetic acid in aqueous solution provided the diol intermediate **9**.

This intermediate represents the key precursor for subsequent syntheses, as the simple workflow makes the process conceivable for subsequent up-scaling. This opens the possibility of producing a wide range of SQDGs with mono-, mixed, or unsaturated fatty acids at the glycerol moiety, starting from the precursor in only a few follow-up steps. In the case presented here, a simple combination with two identical fatty acids to demonstrate the feasibility of the system was carried out exemplarily. However, the preparation of mixed fatty acids by the stepwise introduction of different acyl residues at C-1 and C-2 of glycerol under Steglich conditions at 0 °C is also described in the literature [14,24].

**Scheme 2:**
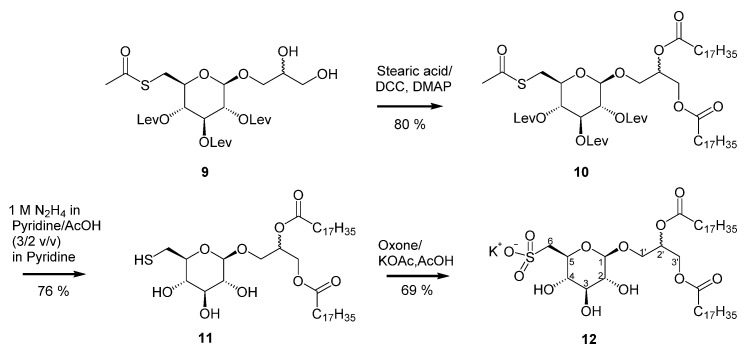
Synthesis of 1,2-distearoyl-3-*O*-[*β*-**d**-sulfoquinovosyl]-(*R*/*S*)-glycerol (SQDG). Conversion of **d**-glucose to the K^+^ form of 1,2-distearoyl-3-*O*-[*β*-**d**-sulfoquinovosyl]-(*R*/*S*)-glycerol with 10% overall yield.

As shown in Scheme 2, the diacylglycerol compound **10** was prepared by condensation of compound **9** with two equivalents of stearic acid under Steglich conditions at room temperature [25]. This was followed by the removal of the levulinyl protecting groups with buffered hydrazine in acetic acid/pyridine solution at room temperature. The use of the levulinyl protecting groups showed advantages compared to former studies. The others used mainly benzyl ether or acetyl groups [10,11,12,13,14]. The removal of levulinyl groups was complete after 30 min at room temperature, when treating an excess of hydrazine. Compared to the use of acetyl groups, it was reported that the removal of these groups was the most difficult step in the synthesis, as it required special tuning of experimental conditions (temperature, time, equivalents) [13,14]. Moreover, difficulties in purification and partial deacylation were reported [14]. In contrast, the byproduct of levunyl removal can be easily separated, because of the water solubility of 4,5-dihydro-6-methyl-3(2*H*) pyridazinone. Partial deacylation of the fatty acid was not observed. Another problem besides the non-specific deacylation that has been reported, when using hydrazine, is the partial hydrogenation of unsaturated fatty acids [8]. However, Cateni et al. (2008) stated that treatment with buffered hydrazine is also applicable in the presence of unsaturated fatty acids [26]. The final step was an oxidation with oxone, leading to the corresponding sulfonic acid, the final SQDG (compound **12**, 69%).

**Scheme 3:**
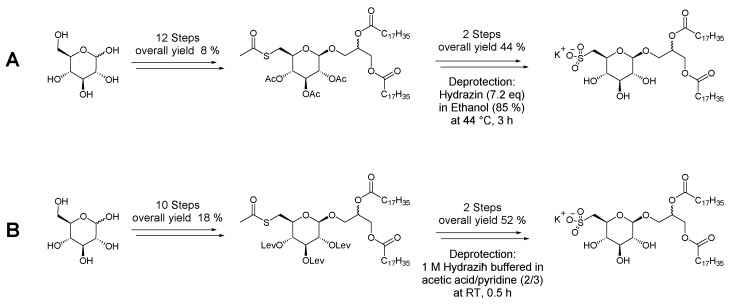
Comparison of the main differences between the synthesis route of (**A**) Manzo et al. (2017) [13] and (**B**) the new route of the present work.

A comparison of the main differences between the new synthesis route and the one presented by Manzo et al. (2017) is shown in Scheme 3. The synthesis route of the present study was shorter by two steps and involved a change in protecting groups in some intermediate steps of the synthesis.

Both routes start with d-glucose and end with the same final product. The route described by Manzo et al. requires a total of 14 steps. The route of this work requires only 12 steps. The shortened route is achieved by direct tosylation, resulting in an overall yield of 10%. In comparison, Manzo et al. (2017) used a sequence of tritylation–acetylation–detritylation–tosylation instead, which reduces the overall yield to 4%. However, the real advantage of the alternative route is the change in protecting group. Manzo et al. used acetyl protecting groups exclusively. This is a disadvantage, because the reaction conditions for the final removal of the acetate protecting group lead to partial cleavage of the fatty acids of the acyl glycerol. When using levulin groups, this problem was not observed due to the milder reaction conditions, which furthermore allowed an easier and faster deprotection.

## 3. Materials and Methods

All reagents were purchased from commercial suppliers (Sigma-Aldrich (Darmstadt, Germany), Honeywell (Seelze, Germany), Acros (Schwerte, Germany) and used without further purification. Solvents (DCM, DMF, pyridine, MeOH, toluene) were purchased as anhydrous over molecular sieves from Acros Organics. Unless otherwise stated, all reactions were performed in dried glassware with magnetic stirring and under nitrogen atmosphere. All reactions were monitored with thin-layer chromatography (TLC) using an aluminum TLC plate (silica gel 60, F_254_, Merck KGaA, Darmstadt, Germany). Components on TLC plates were visualized with cerium-based staining (4.5 g ammonium molybdate tetrahydrate, 0.18 g anhydrous cerium sulfate, 2.5 mL sulfuric acid, and 90 mL H_2_O) or staining with copper (5 g anhydrous copper sulfate, 10 mL o-phosphoric acid 85%, and 90 mL H_2_O) followed by heating. Purification of products was carried out with silica gel 60 (particle size 0.040–0.063 mm, Merck KGaA, Darmstadt, Germany) and freshly distilled solvents were used as eluents. Yields refer to chromatographically purified and spectroscopically pure compounds. NMR spectra were recorded on Bruker instruments (Fourier HD 300; Avance I 400 DRX 500, or Avance III 600 at room temperature. Chemical shift δ is reported in ppm with the solvent resonance signal as the internal standard: chloroform-d1: 7.26 (^1^H NMR), 77.16 (^13^C NMR), methanol-d4: 3.31 (^1^H NMR), 49.00 (^13^C NMR). Coupling constant J is given in Hertz (Hz). Multiplicities are classified as follows: s = singlet, d = doublet, t = triplet, q = quartet, and combinations thereof; m = multiplet or br = broad signal. Two-dimensional NMR experiments (COSY, HSQC, HMBC) were used for the assignment of all NMR signals. The approximately diastereomeric ratio was determined by taking ^1^H and ^13^C NMR data into account. High-resolution mass spectra were recorded on an Agilent 6224 ESI-TOF coupled with an Agilent HPLC 1200 Series. The spectra can be found in the supplementary materials.

**General procedure for preparation of levulinic acid anhydride:** A solution of dicyclohexylcarbodiimide (0.5 equiv.) in dry dichloromethane (15 mL) was cooled to 0 °C. Levulinic acid (1 equiv.) dissolved in dry DCM (10 mL) was added dropwise. The reaction mixture was stirred for 1 h with the formation of a colorless precipitate. The precipitate was removed by filtration and the solvent evaporated in vacuo to result in a colorless syrup. The resulting product was used without further purification.

**1,2,3,4,6-Penta-*O*-acetyl-d-glucopyranose (1):** d-glucose (29.6 g, 0.164 mol) was mixed with 200 mL of anhydrous pyridine. While stirring with a magnetic stirrer, acetic anhydride (240 mL, 2.54 mol) was slowly added. This mixture was kept at room temperature under stirring for 20 h. When the reaction was complete, the solution was mixed with ice and stirred until all ice melted, whereupon a colorless solid precipitated. The mixture was filtered, and the filtered product was washed with ice-cold water to remove pyridine residues. The filtered product was recrystallized in a solution of water/methanol (*v*/*v* = 50/50) and gave compound **1** as a colorless solid (59.6 g, 92%). ^1^H NMR (600 MHz, CDCl_3_): δ 6.33 (d, J = 3.7 Hz, 1H, H-1β), 5.47 (dd, J = 9, J < 1.0 Hz, 1H, H-3), 5.14 (t, J = 9.8 Hz, 1H, H-4), 5.10 (dd, J = 10.3, 3.7 Hz, 1H, H-2), 4.29–4.24 (m, 1H, H-6a), 4.18–4.05 (m, 2H, H-5, H-6b), 2.18, 2.09, 2.04, 2.02, 2.01 (all s, 15H, 5 × COCH_3_). ^13^C NMR (150 MHz, CDCl_3_): δ 171.0, 170.32, 170.28, 169.8, (4 × C=O), 89.2 (C-1α), 69.98 (C-3), 69.97 (C-5) 69.3 (C-2) 68.05 (C-4), 61.6 (C-6), 21.0, 20.83, 20.80, 20.7, 20.6 (5 × C(O)CH_3_).

**2,3,4,6-Tetra-*O*-acetyl-d-glucopyranose (2):** Ammonium acetate (19.8 g, 0.257 mol) was added to a solution of compound **1** (50.0 g, 0.128 mol) in dry N,N-dimethylformamide (100 mL). The solution was stirred at room temperature overnight (30 h). After completion of the reaction (monitored by TLC), the solution was evaporated in vacuo. The residue was purified by silica gel column chromatography (ethyl acetate) to give compound **2** (43.3 g, 97% yield) as an amber-colored syrup. The product was present as an anomeric mixture in a ratio of (α:β) 3:1. ^1^H NMR (500 MHz, CDCl_3_): main signals α-anomer: δ 5.52 (dd, J = 10.2, 9.4 Hz, 1H, H-3), 5.45 (d, J = 3.6 Hz, 1H, H-1β), 5.07 (t, J = 9.7 Hz, 1H, H-4). 4.89 (dd, J = 10.2, 3.6 Hz, 1H, H-2), 4.73 (d, J = 8.0 Hz, 1H, H-1α), 4.26 (td, J = 3.9, 2.0 Hz, 1H, H-5), 4.23—4.20 (m, 1H, H-6a), 4.14–4.09 (m, 1H, H-6b), 2.08, 2.07, 2.02, 2.01 (all s, 12H, 4 × COCH_3_). ^13^C NMR (125 MHz, CDCl_3_): δ 170.8, 170.4, 169.8, 169.5, 168.9 (5 × C=O), 95.7 (C-1 β), 90,3 (C-1 α), 71.2 (C-2), 70.0 (C-3), 68.7 (C-4), 67.4 (C-5), 62,1 (C-6), 20.86, 20.82, 20.80, 20.73 (4 × C(O)CH_3_) HRESIMS *m*/*z*: 371.096 [M + Na]^+^ (calculated for C_14_H_20_O_10_Na, 371.095).

**2,3,4,6-Tetra-*O*-acetyl-d-glucopyranosyl trichloroacetimidate (3):** To a solution of compound **2** (41.9 g, 0.12 mmol) and in dry dichloromethane (120 mL), trichloroacetonitrile (60 mL, 0.6 mol) was added at 0 °C. Then, 1,8-diazabicyclo [5.4.0]undec-7-ene (DBU) (1.8 mL, 12 mmol) was added dropwise into the solution and stirred for 1 h. After the reaction was completed, the reaction mixture evaporated in vacuo. The residue was purified by silica gel column chromatography (ethyl acetate/cyclohexane, *v*/*v*, 50/50) to give compound **2** (48.8 g, 83% yield) as an amber-colored syrup. ^1^H NMR (400 MHz, CDCl_3_): δ 8.69 (s, 1H, NH), 6.55 (d, J = 3.7 Hz, 1H, H-1β), 5.55 (t, J = 9.9 Hz, 1H, H-3), 5.17 (t, J = 9.9 Hz, 1H, H-4), 5.12 (dd, J = 10.2, 3.7 Hz, 1H, H-2), 4.27 (dd, J = 12.3, 4.1 Hz, 1H, H-6a), 4.20 (ddd, J = 10.3, 4.2, 2.1 Hz, 1H, H-5), 4.17–4.08 (m, 1H, H-6b), 2.07, 2.04, 2.02, 2.01 (all s, 12H, 4 × COCH_3_). ^13^C NMR (100 MHz, CDCl_3_): δ 170.7, 170.1, 170.0, 169.6, (4 × C=O), 160.9 (HN=C), 93.0 (C-1), 70.1 (C-5), 70.0 (C-3), 69.8 (C-2), 67.9 (C-4), 61.5 (C-6), 20.80, 20.80, 20.7, 20.56 (4 × C(O)CH_3_). HRESIMS *m*/*z*: 514.002 [M + Na]^+^ (calculated for C_16_H_20_Cl_3_NO_10_Na, 514.005).

**1,2-Isopropylidene-3-*O*-(*β*-d-2,3,4,6-tetra-*O*-acetyl-glucopyranosyl)-(*R*/*S*)-glycerol (4):** To a solution of compound **3** (56.3 g, 0.11 mol) in dry dichloromethane (60 mL), 1,2-*O*-isopropylidene glycerol (26.0 mL, 0.20 mol) was added and cooled to -10 °C. Then, boron trifluoride etherate (1.6 mL, 13 mmol) was added dropwise into the solution and stirred. After 2 h, more boron trifluoride etherate (1.0 mL, 8.1 mmol) was added and slowly warmed until reaching room temperature. After stirring overnight, the reaction mixture was quenched with triethylamine (2 mL) and mixed with ice and stirred until all ice melted, whereupon a beige-colored solid precipitated. The mixture was filtered, and the filtered product was washed with ice-cold water. The filtered product was recrystallized in a solution of water/methanol (*v*/*v*, 50/50) and gave compound **4** as a colorless solid (40.9 g, 80%). The product was present as a diastereomeric mixture in a ratio of 7:3. ^1^H NMR (600 MHz, CDCl_3_): δ 5.19 (tt, J = 9.5, 6.9 Hz, 1H, H-3), 5.09–5.03 (m, 1H, H-4), 5.01–4.95 (m, 1H, H-2), 4.59 (d, J = 8.0 Hz, 1H, H-1α), 4.27–4.22 (m, 1H, H-6a), 4.22–4.19 (m, 1H, H-2′), 4.12 (dd, J = 12.3, 2.4 Hz, 1H, H-6b), 4.02 (dd, J = 8.5, 6.4 Hz, 1H, H-3′a), 3.78 (dd, J = 10.3, 5.9 Hz, 1H, H-1′a), 3.71 (dd, J = 8.4, 5.8 Hz, 1H, H-3′b), 3.68 (dq, J = 7.4, 2.5 Hz, 1H, H-5), 3.63 (dd, J = 10.4, 5.7 Hz, 1H, H-1′b), 2.08, 2.04, 2.01, 1.99 (all s, 12H, 4× COCH_3_), 1.39 (s, 3H, CH_3_), 1.33 (s, 3H, CH_3_). ^13^C NMR (150 MHz, CDCl_3_): δ 170.8, 170.4, 169.51, 169.47, (4× C=O), 109.7 (C(CH_3_)_2_, 100.1 (C-1), 74.5 (C-2′) 72.9 (C-3), 72.0 (C-5), 71.3 (C-2), 70.7 (C-1′), 68.5 (C-4), 66.9 (C-3′), 62.0 (C-6), 26.9 (CH_3_), 25.5 (CH_3_), 20.9, 20.8, 20.73, 20.71 (4× C(O)CH_3_). HRESIMS *m*/*z*: 485.159 [M + Na]^+^ (calculated for C_20_H_30_O_12_Na, 485.163).

**1,2-Isopropylidene-3-*O*-(*β*-d-glucopyranosyl)-(*R*/*S*)-glycerol (5):** Sodium methanolate (3.46 g, 64.1 mmol) was added to a suspension of compound **4** (5.00 g, 10.8 mmol) in dry methanol (50 mL). The reaction mixture was stirred at room temperature. After 2 h, the reaction was completed and the solution was homogeneous. The mixture was neutralized with Amberlyst^®^ 15 (Merck KGaA), filtered, and evaporated in vacuo. The residue was purified by silica gel column chromatography (ethyl acetate/methanol (*v*/*v*, 70/30) to give compound **5** (2.95 g, 92% yield) as a colorless syrup. The product was present as a diastereomeric mixture in a ratio of 7:3. ^1^H NMR (500 MHz, MeOD): 4.36–4.31 (m, 1H, H-2′), 4.29 (d, J = 7.7 Hz, 1H, H-1α), 4.08 (dd, J = 8.5, 6.5 Hz, 1H, H-3′a), 3.90 (dd, J = 10.3, 5.3 Hz, 1H, H-1′a), 3.87 (dd, J = 11.9, 1.7 Hz, 1H, H-6a), 3.77 (dd, J = 8.5, 6.1 Hz, 1H, H-3′b), 3.66 (dd, J = 11.9, 5.3 Hz, 1H, H-6b), 3.62 (dd, J = 10.2, 6.2 Hz, 1H, H-1′b), 3.38–3.33 (m, 1H, H-3), 3.29–3.26 (m, 2H, overlapped H-4, H-5), 3.18 (dd, J = 9.1, 7.7 Hz, 1H, H-2), 1.40 (s, 3H, CH_3_), 1.34 (s, 3H, CH_3_). ^13^C NMR (125 MHz, MeOD): δ 110.6 (C(_CH3_)_2_, 104.5 (C-1), 78.0 overlapped (C-3, C-5), 76.1 (C-2′), 75.1 (C-2), 71.59 (C-1′), 71.57 (C-4), 67.7 (C-3′), 62.7 (C-6), 27.1 (CH_3_), 25.6 (CH_3_), HRESIMS *m*/*z*: 317.121 [M + Na]^+^ (calculated for C_12_H_22_O_8_Na, 317.121).

**1,2-Isopropylidene-3-*O*-[(6-*O*-(4-tolylsulfonyl))-*β*-d-glucopyranosyl]-(*R*/*S*)-glycerol (6):** To a solution of compound **5** (900 mg, 3.06 mmol) in dry pyridine (10 mL), *p*-toluenesulfonyl chloride (TsCl, 654 mg, 3.43 mmol, 1.1 equiv.) was added at 0 °C and stirred overnight at room temperature. An additional amount of TsCl (100 mg, 0.52 mmol) was added. After 2 h, the reaction was quenched with EtOH; solvents were evaporated in vacuo and the residue was purified by flash column chromatography (ethyl acetate/methanol (*v*/*v*, 100:0→90:10)) to give compound 6 (994 mg, 72% yield) as a colorless resin. The product was present as a diastereomeric mixture in a ratio of 7:3. ^1^H NMR (300 MHz, CDCl_3_): δ 7.77 (d, J = 8.3 Hz, 2H, Ar-H), 7.31 (d, J = 8.1 Hz, 2H, Ar-H), 4.35–4.15 (m, 4H, overlapped H-2′, H-1, H-6a,b), 4.01 (dd, J = 8.7, 6.6 Hz, 1H, H-3′a), 3.80–3.71 (m, 1H, H1′a), 3.65 (dd, J = 8.5, 5.8 Hz, 1H, H-3′b), 3.56–3.49 (m, 2H, overlapped H-3, H-1′b), 3.45 3.45–3.37 (m, 2H, overlapped H-4, H-5), 3.34 (t, J = 8.5 Hz, 1H, H-2), 2.42 (s, 3H, aromatic methyl), 1.39 (s, 3H, CH_3_), 1.32 (s, 3H, CH_3_). ^13^C NMR (75 MHz, CDCl_3_): δ 145.1, Ar, (CCH_3_), 132.4 Ar, (CSO3), 130.0, 128.1 (4× CH, aromatic methynes), 109.9 (C(CH_3_)_2_, 95.8 (C-1), 76.1 (C-3), 74.7 (C-2′), 73.6 (C-5), 73,3 (C-2), 71.3 (C-1′), 69.7 (C-4), 69.2 (C-6), 66.6 (C-3′), 27.1 (CH_3_), 25.6 (CH_3_), 21.2 (Ar, CCH_3_), HRESIMS *m*/*z*: 471.131 [M + Na]^+^ (calculated for C_19_H_28_O_10_SNa, 471.130).

**1,2-Isopropylidene-3-*O*-[(2′,3′,4′-tri-*O*-levulinyl-6′-*O*-(4-tolylsulfonyl))-*β*-d-glucosyl]-(*R*/*S*)-glycerol: (7):** To a solution of compound **6** (990 mg, 2.01 mmol) in dry pyridine (10 mL), levulinic anhydride (3.0 mL, 15 mmol) dissolved in dry pyridine (7 mL) was added dropwise. The reaction was stirred overnight at room temperature. After the reaction was complete, ice water (50 mL) was added. The mixture was extracted with chloroform (3 × 50 mL) and the combined organic layers were extracted with a 10% solution of sodium bicarbonate (2 × 50 mL) first and then with water, dried over Na_2_SO_4_, and concentrated in vacuo. The residue was purified by silica gel column chromatography (ethyl acetate/cyclohexane (*v*/*v*, 50/50→100/0)) to give 7 (1.42 g, 87%) as a colorless resin. The product was present as a diastereomeric mixture in a ratio of 7:3. ^1^H NMR (400 MHz, CDCl_3_): δ 7.79 (d, J = 8.4 Hz, 2H, Ar-H), 7.34 (d, J = 8.2 Hz, 2H, Ar-H), 5.21 (t, J = 9.6 Hz, 1H, H-3), 4.90 (m, 2H, overlapped H-2, H-4), 4.51 (d, J = 7.9 Hz, 1H, H-1α), 4.24–4.18 (m, 1H, H-2′), 4.15 (dd, J = 11.6, 2.8 Hz, 1H, H-6a), 4.04 (dd, J = 11.6, 6.5 Hz, 1H, H-6b), 4.00 (d, J = 6.5 Hz, 1H, H-3′a), 3.80–3.69 (m, 2H, overlapped H-5, H-1′a), 3.66 (dd, J = 8.5, 5.9 Hz, 1H, H-3′b), 3.57 (dd, J = 10.5, 5.4 Hz, 1H, H-1′b), 2.82–2.66 (m, 6H, 3 × CH_2_OCO), 2.64–2.46 (m, 6H, 3 × CH_2_COCH_3_), 2.44 (s, 3H, aromatic methyl), 2.15 (s, 3H), 2.15 (s, 3H), 2.13 (s, 3H), (3 × CH_3_CO), 1.39 (s, 3H, CH_3_), 1.33 (s, 3H, CH_3_). ^13^C NMR (100 MHz, CDCl_3_): δ 206.37, 206.34, 206.31 (3 × CH_3_CO), 172.1, 171.7, 171.3 (3 × CH_2_OCO), 145.2, (Ar, CCH_3_), 132.8 (Ar, CSO_3_), 130.0, 128.2 (4 × CH, aromatic protons), 109.7 (C(CH_3_)_2_, 100.8 (C-1), 74.5 (C-2′), 72.0 (overlapped C-3, C-5), 71.01 (C-2), 70.7 (C-1′), 68.6 (C-4), 68.0 (C-6), 66.9 ( C-3′) 37.90, 37.84, 37.80 (3 × CH_2_COCH_3_), 29.86, 29.79, 29.77, (3 × CH_3_CO), 27.91, 27.88, 27.83 (3 × CH_2_OCO), 26.9 (CH_3_), 25,5 (CH_3_), 21.8 (Ar, CCH_3_), HRESIMS *m*/*z*: 765.239 [M + Na]^+^ (calculated for C_34_H_46_O_16_SNa, 765.240).

**1,2-Isopropylidene-3-*O*-[(2′,3′,4′-tri-*O*-levulinyl-6′-thioacetyl)-*β*-d-glucosyl]-(*R*/*S*)-glycerol: (8)** Compound **7** (1.40 g, 1.88 mmol) was dissolved in dry dimethylformamide (10 mL) and potassium thioacetate (646 mg, 5.66 mmol) was added. The reaction was stirred at 80 °C. After 2 h, the reaction mixture was cooled until reaching room temperature and further stirred overnight. Subsequently, the solvents were evaporated in vacuo and the residue was purified by silica gel column chromatography (ethyl acetate) to give compound **8** (926 mg, 76% yield) as a colorless resin. The product was present as a diastereomeric mixture in a ratio of 7:3. ^1^H NMR (400 MHz, CDCl_3_): δ 5.20 (t, J = 9.7 Hz, 1H, H-3), 5.00–4.88 (m, 2H, overlapped H-2, H-4), 4.52 (d, J = 8.0 Hz, 1H, H-1α), 4.23 (p, J = 5.9 Hz, 1H, H-2′), 4.03 (dd, J = 8.4, 6.4 Hz, 1H, H-3′a), 3.85–3.68 (m, 2H, overlapped H-1′a, H-3′b), 3.63 (dd, J = 10.4, 5.7 Hz, 1H, H-1′b), 3.60–3.53 (m, 1H, H-5), 3.28 (dd, J = 14.3, 3.0 Hz, 1H, H-6a), 3.01–2.92 (m, 1H, H-6b), 2.81–2.68 (m, 6H, 3 × CH_2_OCO), 2.63–2.50 (m, 6H, 3 × CH_2_COCH_3_), 2.32 (s, 3H, CH_3_C(O)S), 2.16 (s, 3H), 2.15 (s, 3H), 2.13 (s, 3H) (3 × CH_3_CO), 1.39 (s, 3H, CH_3_), 1.33 (s, 3H, CH_3_). ^13^C NMR (100 MHz, CDCl_3_): δ 206.6, 206.5, 206.4 (3 × CH_3_CO), 194.8 (CH_3_C(O)S), 172.1, 171.8, 171.4 (3 × CH_2_OCO), 109.6 (C(CH_3_)_2_, 100.8 (C-1), 74.5 (C-2′), 73.3 (C-5), 72.1 (C-3), 71.3 (C-4), 70.8 (C-2), 70.5 (C-1′) 67.0 (C-3′), 37.93, 37.87, 37.84 (3 × CH_2_COCH_3_), 30.5 (CH_3_C(O)S), 30.2 (C-6), 29.9, 29.8, 29.79 (3 × CH_3_CO), 28.02, 27.95, 27.93 (3 × CH_2_OCO), 26.9 (CH_3_), 25.5 (CH_3_). HRESIMS *m*/*z*: 669.219 [M + Na]^+^ (calculated for C_29_H_42_O_14_SNa, 669.219).

**3-O-[(2′,3′,4′-tri-*O*-levulinyl-6′-thioacetyl)-*β*-d-glucosyl]-(*R*/*S*)-glycerol (9):** A solution of compound **8** (876 mg, 1.35 mmol) in 80% aq acetic acid (10 mL) was stirred at 45 °C for 2 h. The mixture was evaporated in vacuo and co-evaporated with toluene three times. The residue was purified by silica gel column chromatography (ethyl acetate/methanol (*v*/*v*, 100/0→70/30)) to give 9 (730 mg, 89%) as a colorless resin. The product was present as a diastereomeric mixture in a ratio of 7:3. ^1^H NMR (300 MHz, CDCl_3_): δ 5.22 (t, J = 9.7 Hz, 1H, H-3), 5.01–4.89 (m, 2H, overlapped H-2, H-4), 4.48 (d, J = 8.0 Hz, 1H, H-1α), 3.96–3.80 (m, 2H, overlapped H-2′, H-1′a), 3.72–3.50 (m, 4H, overlapped H-1′b, H-3′a, H-3′b, H-5), 3.29 (dd, J = 14.3, 2.7 Hz, 1H, H-6a), 2.96 (dd, J = 14.3, 7.2 Hz, 1H, H-6b), 2.86–2.67 (m, 6H, 3 × CH_2_OCO), 2.64–2.47 (m, 6H, 3 × CH_2_COCH_3_), 2.33 (s, 3H, CH_3_C(O)S), 2.18 (s, 3H), 2.16 (s, 3H), 2.13 (s, 3H) (3 × CH_3_CO). ^13^C NMR (75 MHz, CDCl_3_): δ 208.1, 206.7, 206.5 (3 × CH_3_CO), 195.0 (CH_3_C(O)S), 172.0, 171.8, 171.6 (3 × CH_2_OCO), 101.4 (C-1), 73.2 (C-5), 72.2 (C-1′), 72.0 (C-3), 71.3 (C-4), 70.7 (C-2), 70.6 (C-2′), 63.4 (C-3′), 38.0, 37.9, 37.8 (3 × CH_2_COCH_3_), 30.6 (CH_3_C(O)S), 30.1 (C-6), 29.9, 29.81, 29.80 (3 × CH_3_CO), 28.0, 27.9, 27.8 (3 × CH_2_OCO). HRESIMS *m*/*z*: 629.188 [M + Na]^+^ (calculated for C_26_H_38_O_14_SNa, 629.188).

**1,2-Distearoyl-3-*O*-[(2′,3′,4′-tri-*O*-levulinyl-6′-thioacetyl)-*β*-d-glucosyl]-(*R*/*S*)-glycerol (10):** To a solution of compound **9** (700 mg, 1.15 mmol) in dry dichloromethane (15.0 mL), stearic acid (854 mg, 3.00 mmol), dicyclohexylcarbodiimide (608 mg, 2.90 mol), and 4-(Dimethylamino)-pyridine (67 mg, 0.5 mmol) were successively added. The reaction mixture was stirred overnight for 24 h at room temperature. The precipitate was removed by filtration and the solvent evaporated in vacuo. The residue was purified by silica gel column chromatography eluted with ethyl acetate/cyclohexane, *v*/*v*, 50/50 to give the product **10** (1.05 g, 80%) as a colorless waxy solid. The product was present as a diastereomeric mixture in a ratio of 7:3. ^1^H NMR (400 MHz, CDCl_3_): δ 5.23–5.13 (m, 2H, overlapped H-3, H-2′), 4.98–4.91 (m, 2H, overlapped H-2, H-4), 4.45 (d, J = 8.0 Hz, 1H, H-1α), 4.28 (dd, J = 12.0, 3.7 Hz, 1H, H-3′a), 4.07 (dd, J = 12.0, 6.4 Hz, 1H, H-3′b), 3.89 (dd, J = 10.9, 5.3 Hz, 1H, H-1′a), 3.65 (dd, J = 10.9, 5.4 Hz, 1H, H-1′b), 3.57 (ddd, J = 10.0, 7.2, 2.9 Hz, 1H, H-5), 3.28 (dd, J = 14.3, 2.9 Hz, 1H, H-6a), 2.96 (dd, J = 14.3, 7.2 Hz, 1H, H-6b), 2.87–2.67 (m, 6H, 3 × CH_2_OCO), 2.66–2.47 (m, 6H, 3 × CH_2_COCH_3_), 2.33 (s, 3H, CH_3_C(O)S), 2.29 (dd, J = 9.0, 6.3 Hz, 4H, α-methylene of stearoyl), 2.16 (s, 3H), 2.15 (s, 3H), 2.13 (s, 3H) (3 × CH_3_CO), 1.63–1.54 (m, 4H, β-methylene of stearoyl), 1.18 (br s, 56H, aliphatic methylenes of stearoyl), 0.81 (t, J = 7.2, 6.4 Hz, 6H, 2 × terminal CH_3_ of stearoyl). ^13^C NMR (100 MHz, CDCl_3_): δ 206.7, 206.5, 206.4 (3 × CH_3_CO), 194.7 (CH_3_C(O)S), 173.5, 173.2 (2 × C(O)O of stearoyl), 172.1, 171.8, 171.3 (3 × CH_2_OCO), 100.7 (C-1), 73.3 (C-5), 72.0 (C-3), 71.1 (C-4), 70.6 (C-2), 69.9 (C-2′), 67.6 (C-1′), 62.4 (C-3′), 37.94, 37.87, 37.84 (3 × CH_2_COCH_3_), 34.4, 34.2 (2 × αC -methylene), 30.6 (CH_3_C(O)S), 30.6 (C-6), 30.9, 29.98, 29.94 (overlapped 3 × CH_3_CO), 32.1, 29.9–29.2, 22.8 (aliphatic methylenes, in part overlapped), 28.0, 27.9, 27.8 28.0, 27.9, 27.8 (3 × CH_2_OCO), 25.0 (2 × βC-methylene of stearoyl), 14.3 (2 × terminal CH_3_ of stearoyl). HRESIMS *m*/*z*: 1183.757 [M + HCOO^−^]^−^ (calculated for C_63_H_108_O_18_^−^, 1183.718).

**1,2-Distearoyl-3-*O*-[(6′-thiol)-*β*-d-glucosyl]-(R/S)-glycerol (11):** To a solution of compound **10** (500 mg, 0.44 mmol) in dry pyridine (5 mL), 1 M hydrazine monohydrate in pyridine/glacial acetic acid (*v*/*v*, 3/2) (10 mL) was added. The reaction mixture was stirred for 30 min at room temperature. After the reaction was completed, ice water (50 mL) was added. The mixture was extracted with chloroform (3 × 50 mL) and the combined organic layers were extracted with a 10% solution of sodium bicarbonate (2 × 50 mL) first and then with water, dried over Na_2_SO_4_, and concentrated in vacuo. The residue was purified by silica gel column chromatography (chloroform/methanol, *v*/*v*, 97/3→92/8) to give compound **11** (270 mg, 76%) as a colorless solid. The product was present as a diastereomeric mixture in a ratio of 7:3. ^1^H NMR (300 MHz, CDCl_3_): δ 5.34–5.16 (m, 1H, H-2′), 4.42 (dd, J = 12.0, 3.9 Hz, 1H, H-3′a), 4.29 (d, J = 7.7 Hz, 1H, H-1α), 4.14 (dd, J = 12.0, 6.2 Hz, 1H, H-3′b), 3.98 (dd, J = 11.0, 5.5 Hz, 1H, H-1′a), 3.70 (dd, J = 11.0, 5.9 Hz, 1H, H-1′b), 3.58–3.45 (m, 2H, overlapped H-3, H-4), 3.41–3.34 (m, 2H, overlapped H-2, H-5), 2.97 (ddd, J = 14.1, 9.5, 2.7 Hz, 1H, H-6a), 2.74 (dt, J = 14.3, 7.4 Hz, 1H, H-6b), 2.40–2.23 (m, 4H, α-methylene of stearoyl), 1.66–1.52 (m, 4H, β-methylene of stearoyl), 1.37–1.19 (m, 56H, aliphatic methylenes of stearoyl), 0.88 (t, J = 6.5 Hz, 6H, 2 × terminal CH_3_ of stearoyl). ^13^C NMR (75 MHz, CDCl_3_): δ 173.9, 173.5 (2 × C(O)O of stearoyl), 103.1 (C-1), 76.33 (C-3), 76.30 (C-5), 73.8 (C-2), 72.2 (C-4), 70.10 (C-2′), 68.0 (C-1′), 62.5 (C-3′), 34.5, 34.3 (2 × αC-methylene), 32.1, 29.9–29.2, 22.8 (aliphatic methylenes, overlapped), 26.4 (C-6), 25.1, 25.0 (2 × βC-methylene), 14.3 (2 × terminal CH_3_ of stearoyl), HRESIMS *m*/*z*: 801.580 [M − H]^-^ (calculated for C_45_H_85_O_9_S^−^, 801.592).

**1,2-Distearoyl-3-*O*-[*β*-d-sulfoquinovosyl]-(*R*/*S*)-glycerol-potassium salt (12)**: To a solution of compound **11** (170 mg, 0.211 mmol) in glacial acetic acid (5 mL), potassium acetate (550 mg, 5.60 mmol) and oxone (2 KHSO_5_, KHSO_4_, K_2_SO_4_) (560 mg, 1.82 mmol, 4 equiv. KHSO_5_) were added. The resultant mixture was stirred at room temperature for 24 h. After the reaction was complete, the mixture was evaporated in vacuo and the residue purified by flash chromatography (chloroform/methanol/water, *v*/*v*/v, 92/8/0→65/25/4) to give compound **12** (120 mg 69%), as a colorless solid. The product was present as a diastereomeric mixture in a ratio of 7:3. ^1^H NMR (300 MHz, CDCl_3_): δ 5.30–5.21 (m, 1H, H-2′), 4.43 (dd, J = 12.1, 3.1 Hz, 1H, H-3′a), 4.28 (d, J = 7.7 Hz, 1H, H-1α), 4.14 (dd, J = 12.1, 6.8 Hz, 1H, H-3′b), 4.01 (dd, J = 11.0, 5.3 Hz, 1H, H-1′a), 3.77–3.66 (m, 2H, overlapped H-1′b, H-5), 3.39–3.36 (m, 1H, H-3), 3.36–3.33 (m, 1H, H-6a), 3.26–3.21 (m, 1H, H-2), 3.20–3.16 (m, 1H, H-4), 3.04 (dd, J = 14.4, 7.3 Hz, 1H, H-6b), 2.30 (td, J = 7.5, 5.0 Hz, 4H, α-methylene of stearoyl), 1.65–1.52 (m, 4H, β-methylene of stearoyl), 1.24 (br s, 56H, aliphatic methylenes of stearoyl), 0.86 (t, J = 7.2, 6.6 Hz, 6H, 2 × terminal CH_3_ of stearoyl). ^13^C NMR (75 MHz, CDCl_3_): δ 174.5, 174.2 (2 × C(O)O of stearoyl), 103.5 (C-1), 76.4 (C-3), 73.9 (C-2), 73.6 (C-4), 72.4 (C-5), 70.7 (C-2′), 68.2 (C-1′), 63.2 (C-3′), 54.0 (C-6), 34.6, 34.4 (2 × αC-methylene), 32.2, 30.0–29.4, 22.8 (aliphatic methylenes, in part overlapped), 25.2 (2 × βC-methylene of stearoyl), 14.3 (2 × terminal CH_3_ of stearoyl). HRESIMS *m*/*z*: 849.562 [M − H]^−^ (calculated for C_45_H_85_O_12_S^−^, 849.577).

## 4. Conclusions

The pharmacological and physiological activity of micronutrients such as SQDGs is of increasing interest. Therefore, new applications are needed for analyzing these minor components. However, there are challenges in isolating the individual products and obtaining reference substances of sufficient purity to evaluate bioactivity. SQDGs can only be extracted from natural sources as a mixture of lipoformes and, therefore, require methods for synthesis. Consequently, an alternative approach was proposed, inspired by previous studies, but using a new protecting group strategy that allows simple and selective deprotection. The proposed synthesis route was primarily developed for preparing different SQDGs as reference compounds. This synthesis did not aim at enantiomerically pure components to keep it as simple as possible. However, when applying these compounds as reference substances in food analysis, determination of single diastereomers might only play a minor role. Nonetheless, further experiments could be used to adapt synthesis for enantiomerically pure products, e.g., for research questions or pharmacological applications. The synthesis presented herein achieved an overall yield of 10% and opens up possibilities for a wide range of SQDGs, containing mono-, mixed, or unsaturated fatty acids.

## Data Availability

The data sets presented in this study are available on request from the corresponding author.

## Supplementary Materials

The original ^1^H and ^13^C NMR spectra are available in the supplementary material.
