# Supplementary material for: An Alternative Approach for the Synthesis of Sulfoquinovosyldiacylglycerol
Source: Molecules. 2021 Jul 14;26(14):4275. doi: 10.3390/molecules26144275 (PMC8307113; doi:10.3390/molecules26144275)
Supplement: Supplementary file 1 [file molecules-26-04275-s001.zip › molecules-1283310-supplementary.pdf]

Supporting Information

An alternative approach for the synthesis of Sulfoquinovosyldiacylglycerol

Tobias Sitz[1], Hendrik Domey[1], Judith Fischer[1], Sascha Rohn[1,2,*]

Supporting Information

NMR spectra of the synthesized compounds S3-S14

1,2,3,4,6-Penta-*O*-acetyl-D-glucopyranose (1):

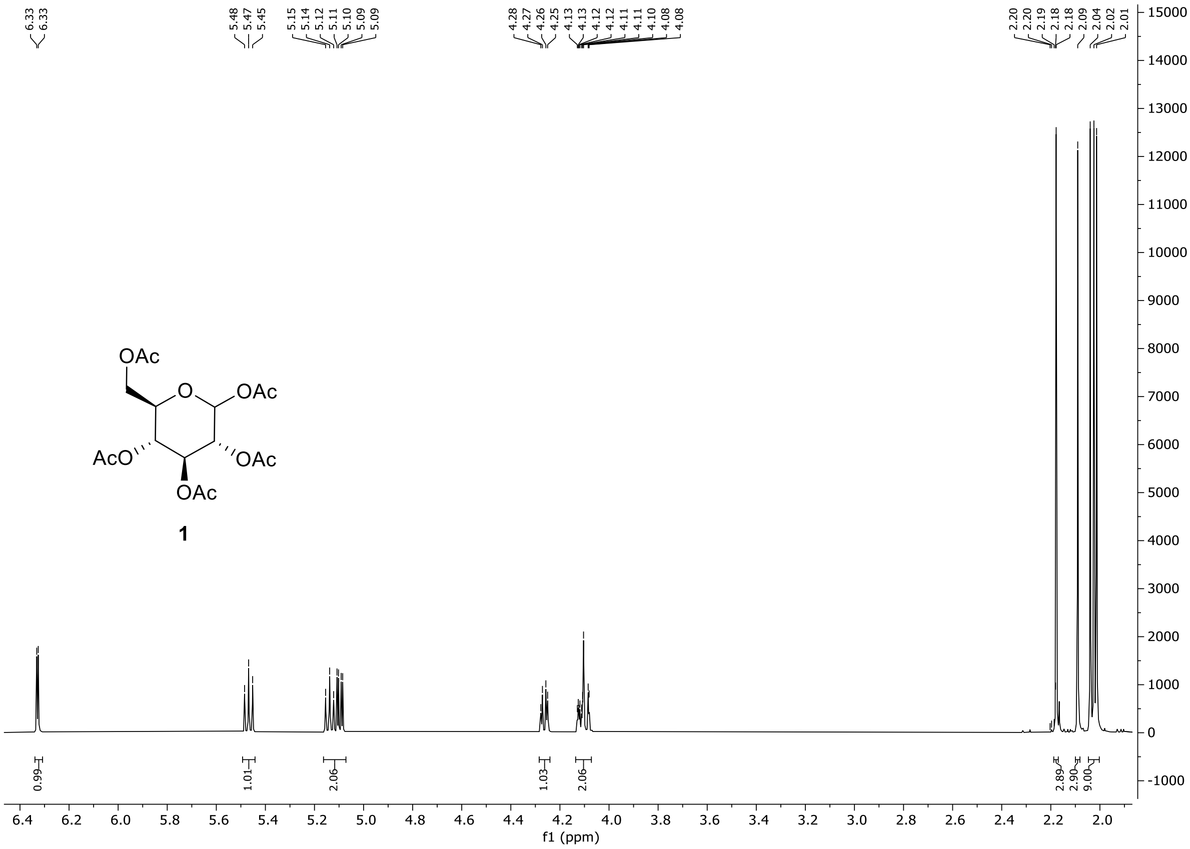

¹H-NMR spectrum (600 MHz, CDCl₃)

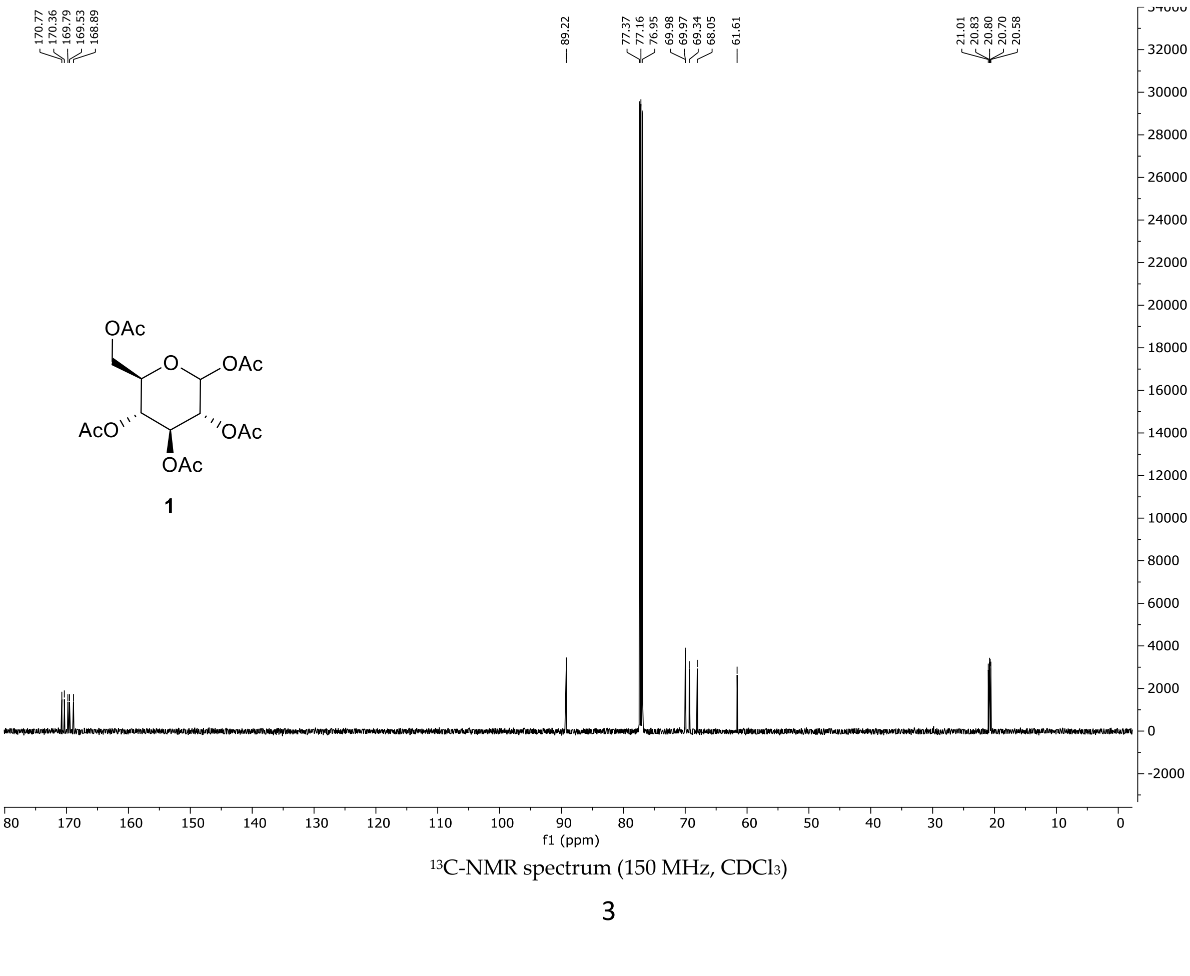

^{13}C-NMR spectrum (150 MHz, CDCl$_3$)

2,3,4,6-Tetra-*O*-acetyl-D-glucopyranose (2):

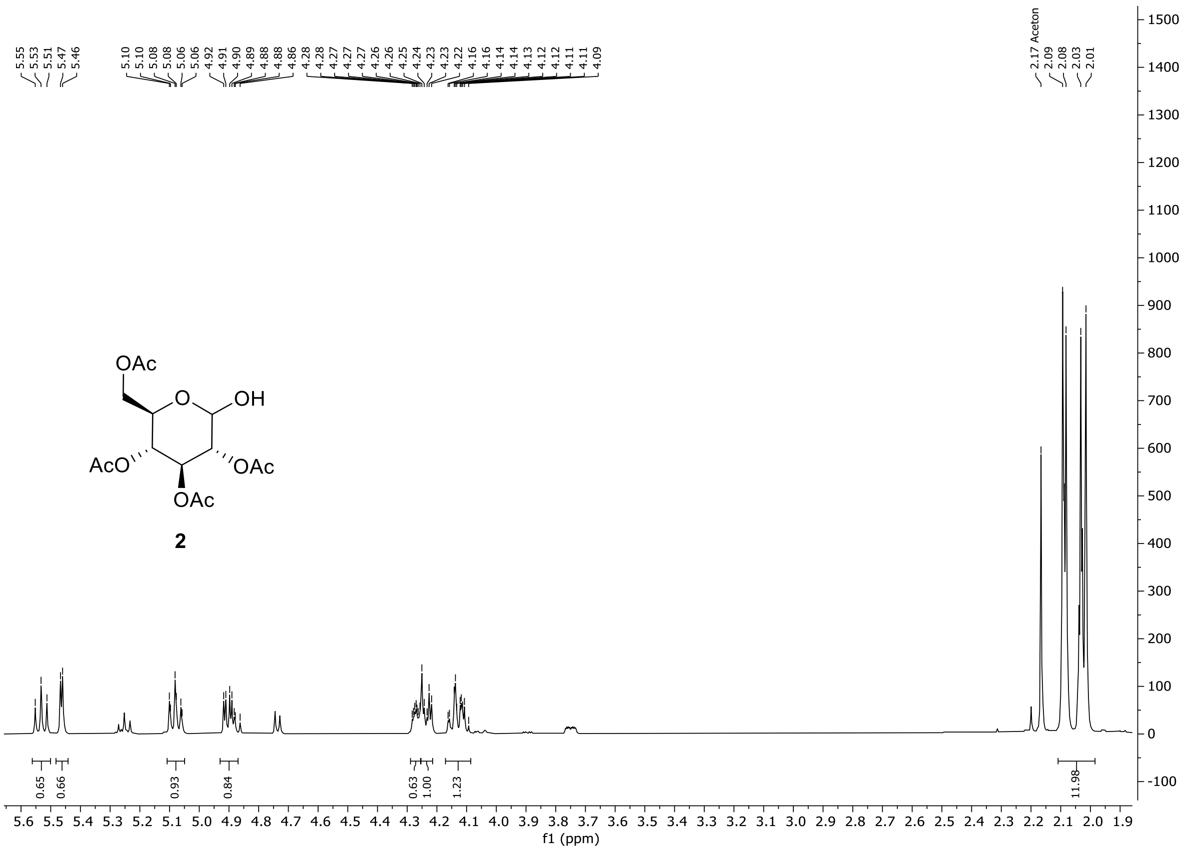

^{1}H-NMR spectrum (500 MHz, $CDCl_3$)

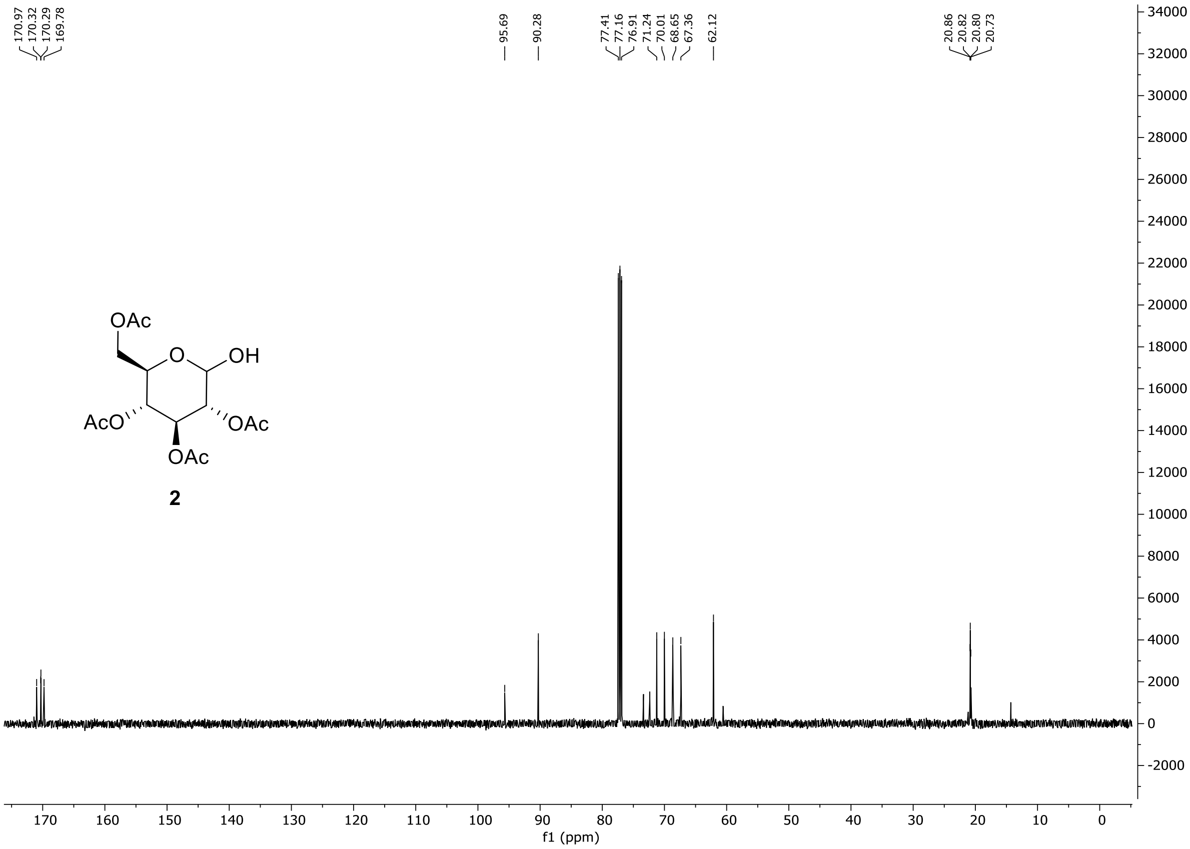

^{13}C-NMR spectrum (125 MHz, $CDCl_3$)

2,3,4,6-Tetra-*O*-acetyl-D-glucopyranosyl trichloroacetimidate (3):

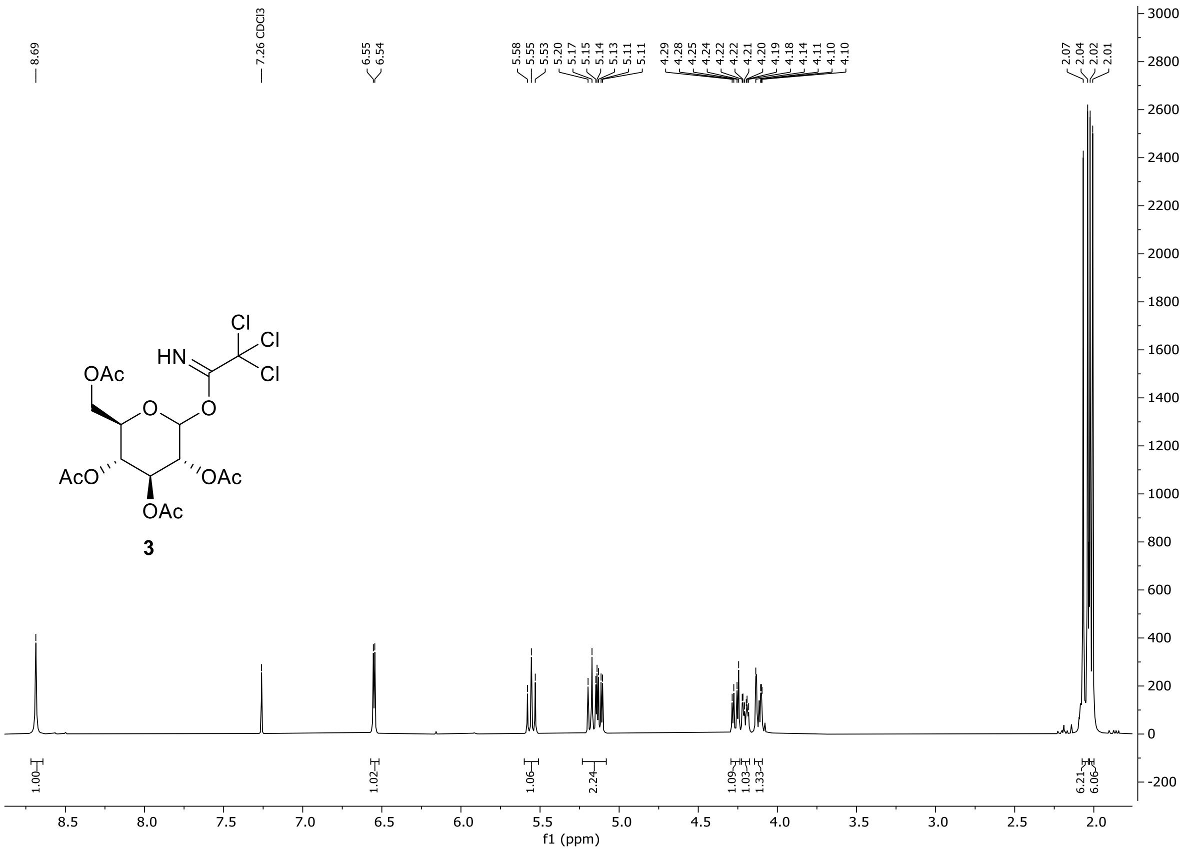

^{1}H-NMR spectrum (400 MHz, CDCl$_3$)

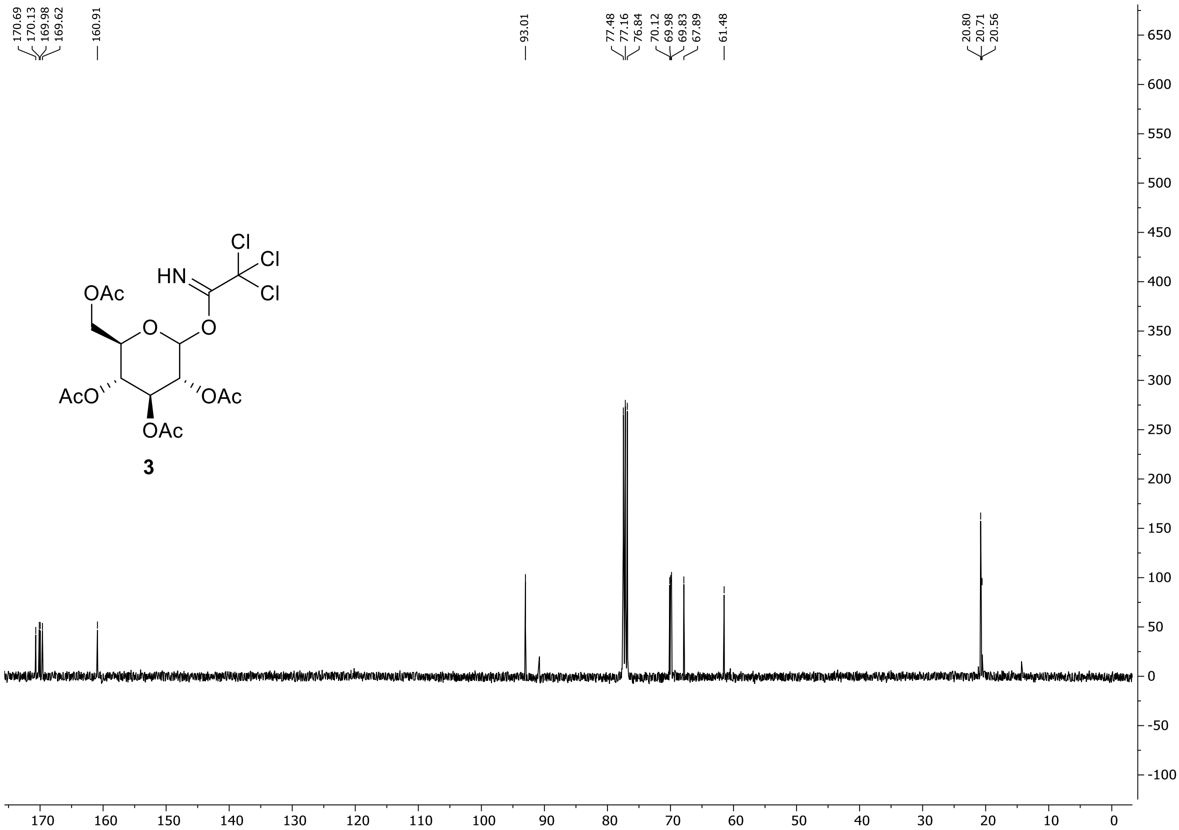

^{13}C-NMR spectrum (100 MHz, CDCl$_3$)

1,2-Isopropylidene-3-*O*-(β-D-2,3,4,6-tetra-*O*-acetyl-glucopyranosyl)-(R/S)-glycerol (4):

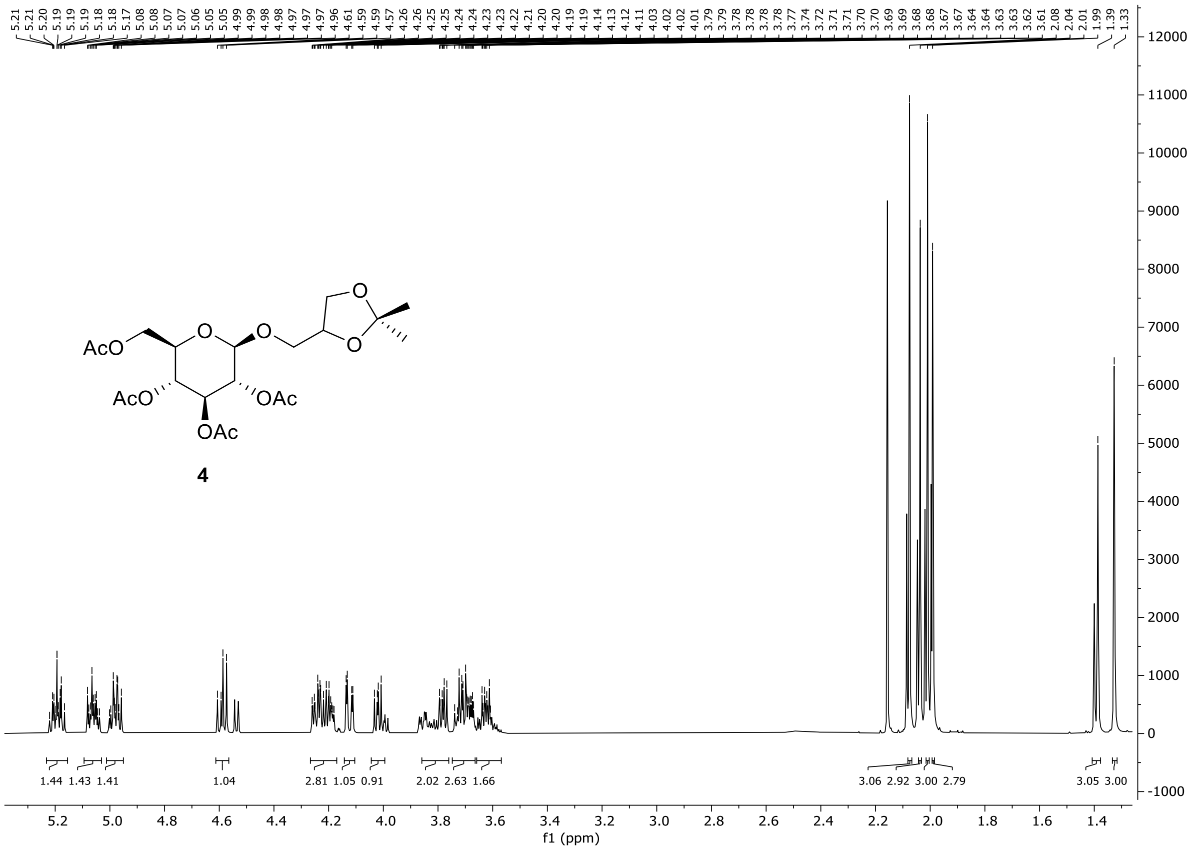

^{1}H-NMR spectrum (600 MHz, $CDCl_3$)

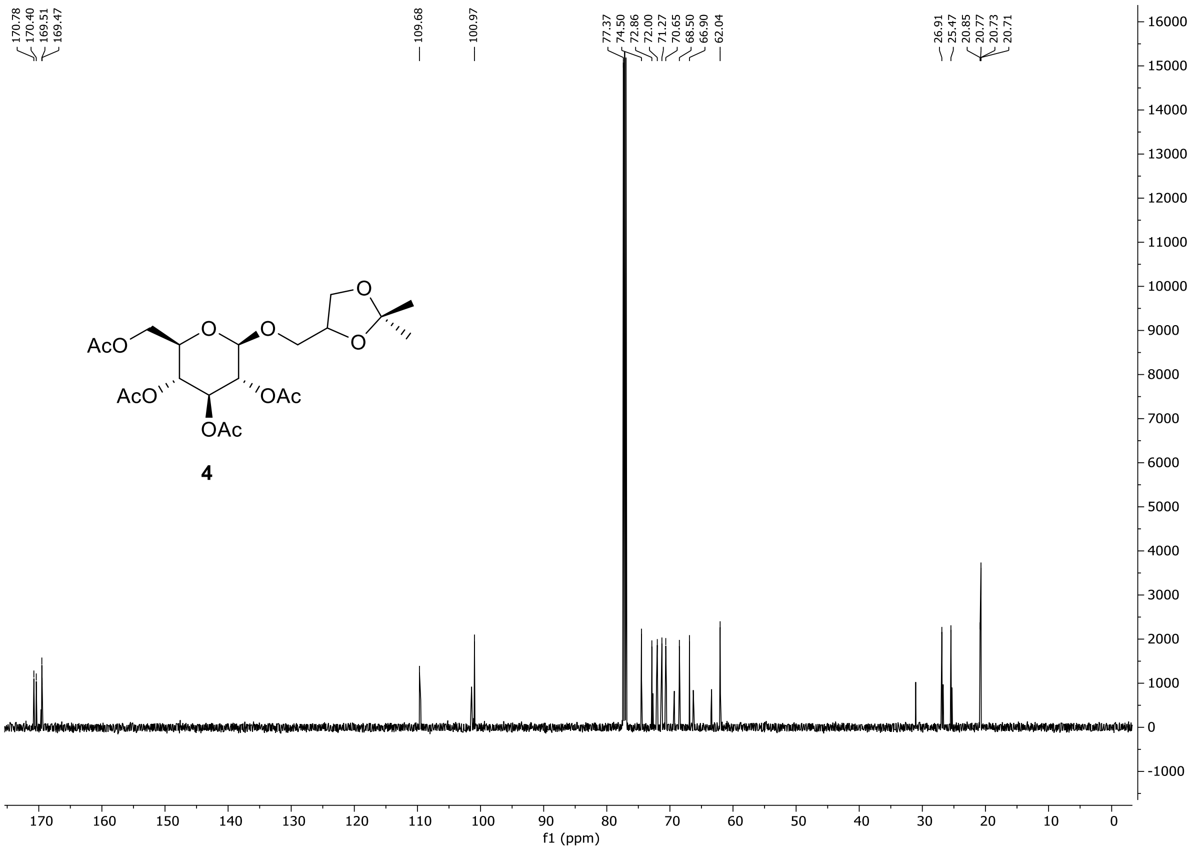

^{13}C-NMR spectrum (150 MHz, $CDCl_3$)

1,2-Isopropylidene-3-*O*-(β-D-glucopyranosyl)-(R/S)-glycerol (5):

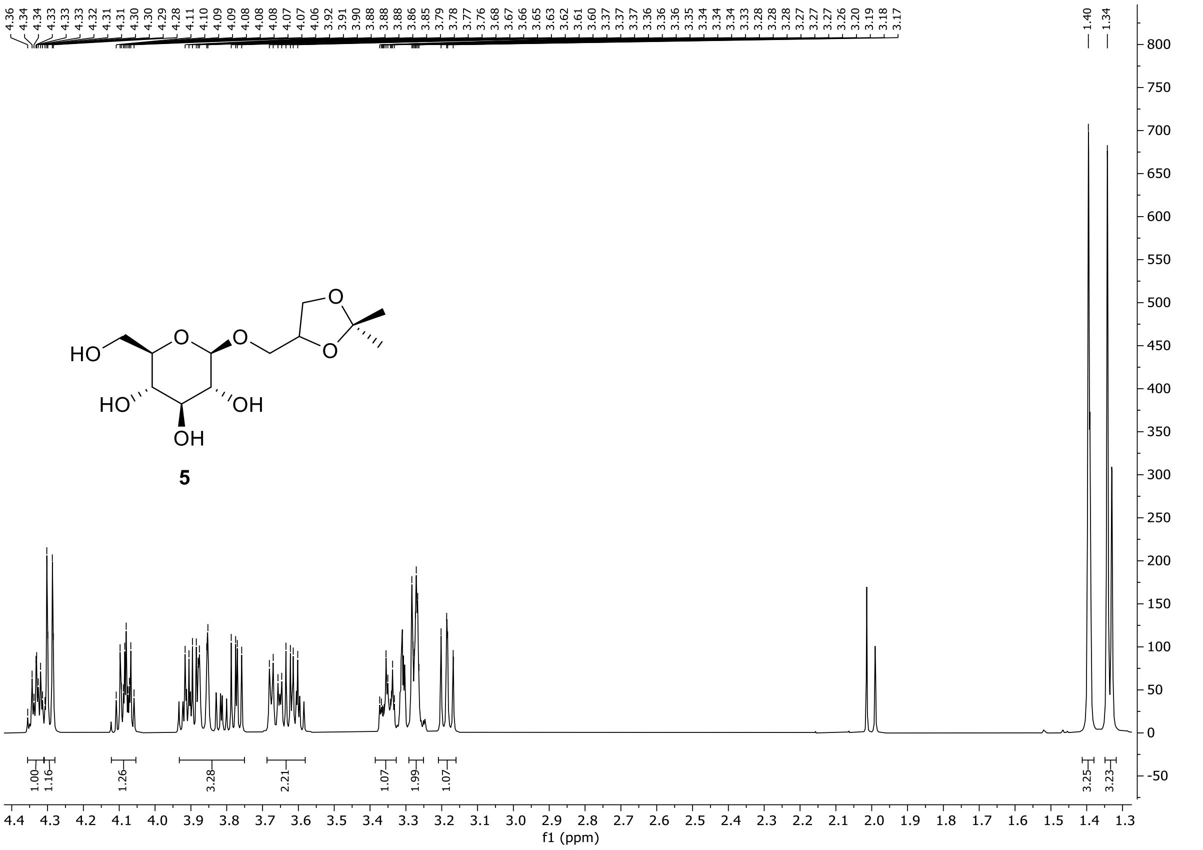

^{1}H-NMR spectrum (500 MHz, MeOD)

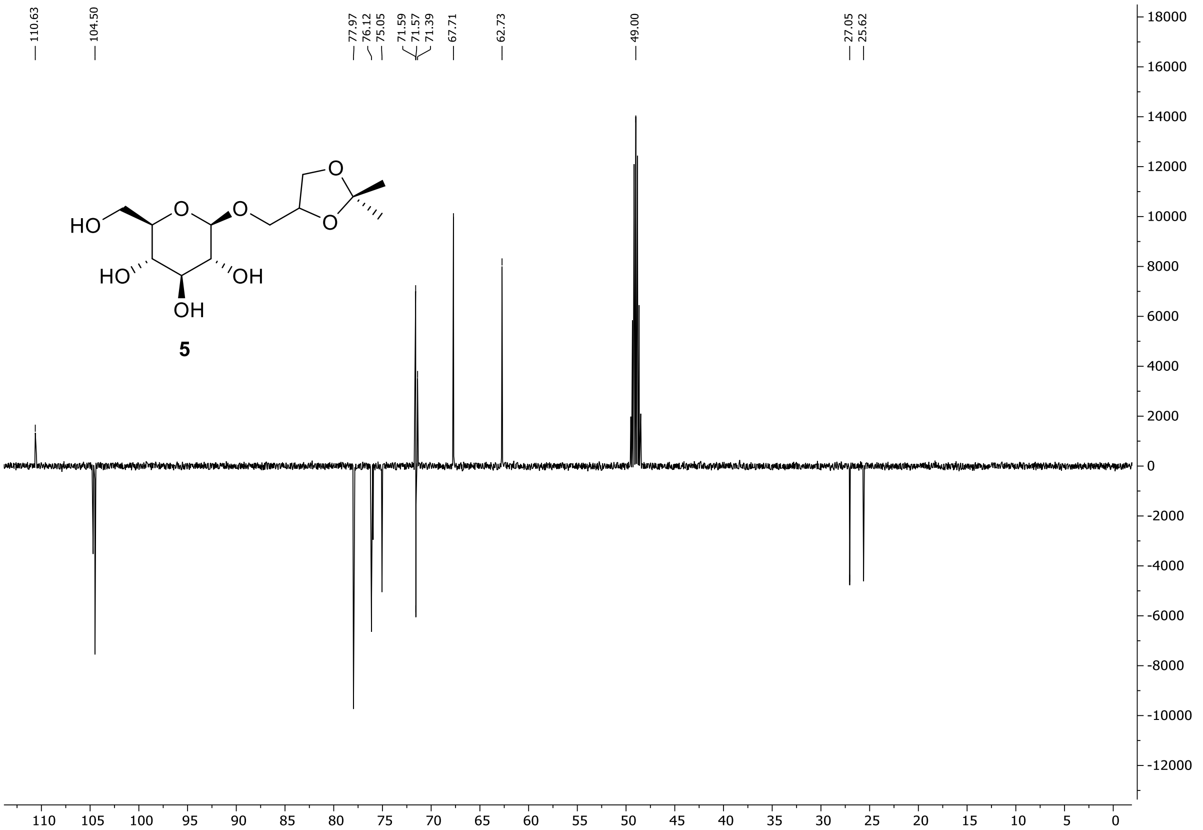

^{13}C- DEPTQ 135 NMR spectrum (125 MHz, MeOD)

1,2-Isopropylidene-3-*O*-[(6-*O*-(4-tolylsulfonyl))-β-D-glucopyranosyl]-(R/S)-glycerol (6):

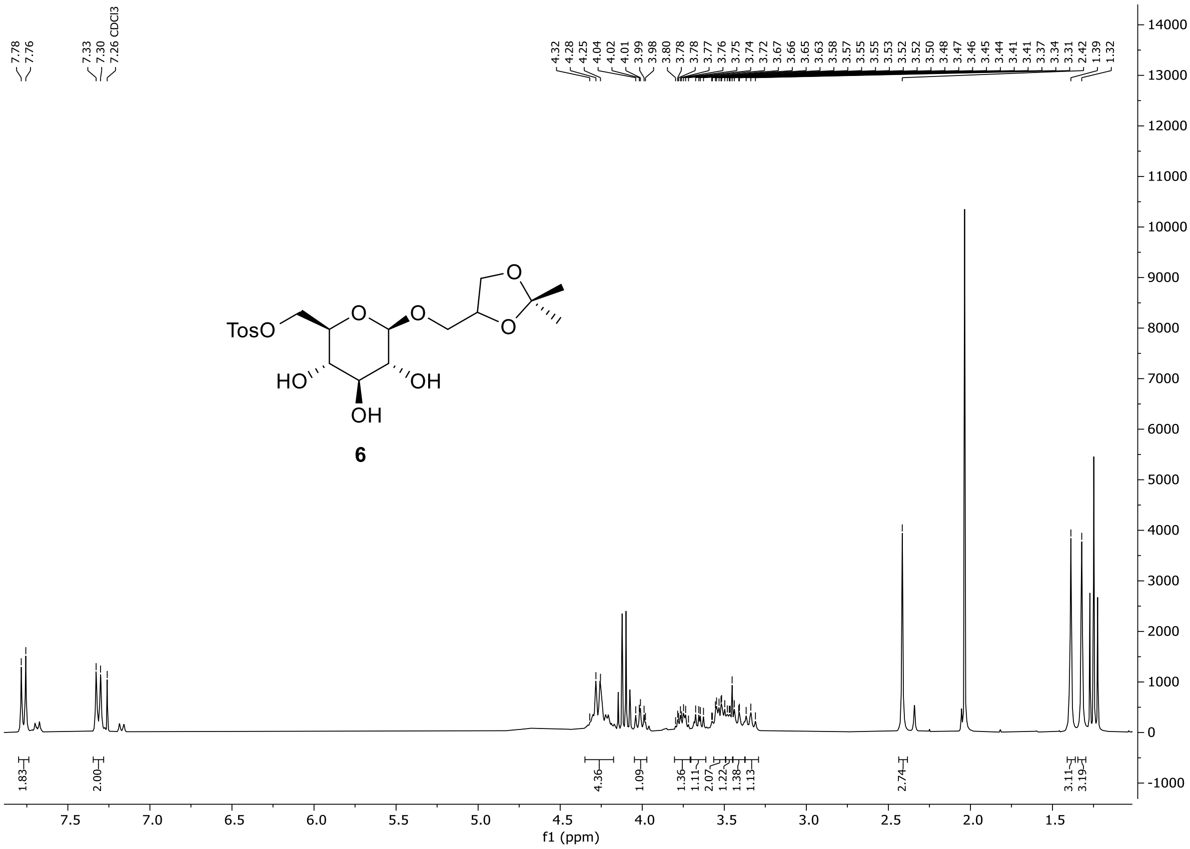

^{1}H-NMR spectrum (500 MHz, $CDCl_3$)

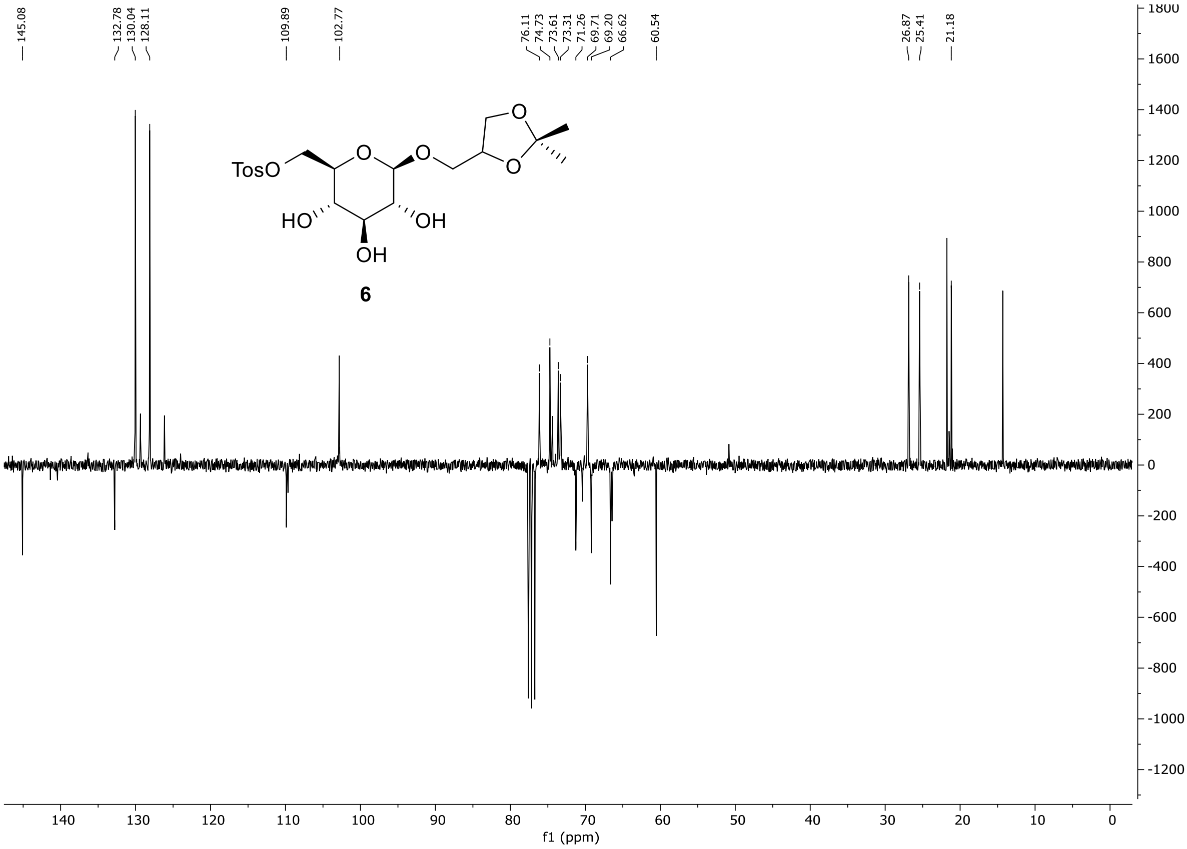

^{13}C- DEPTQ 135 NMR spectrum (125 MHz, $CDCl_3$)

1,2-Isopropylidene-3-*O*-[(2′,3′,4′-tri-*O*-levulinyl-6′-*O*-(4-tolylsulfonyl))-β-D-glucosyl]-(R/S)-glycerol: (7):

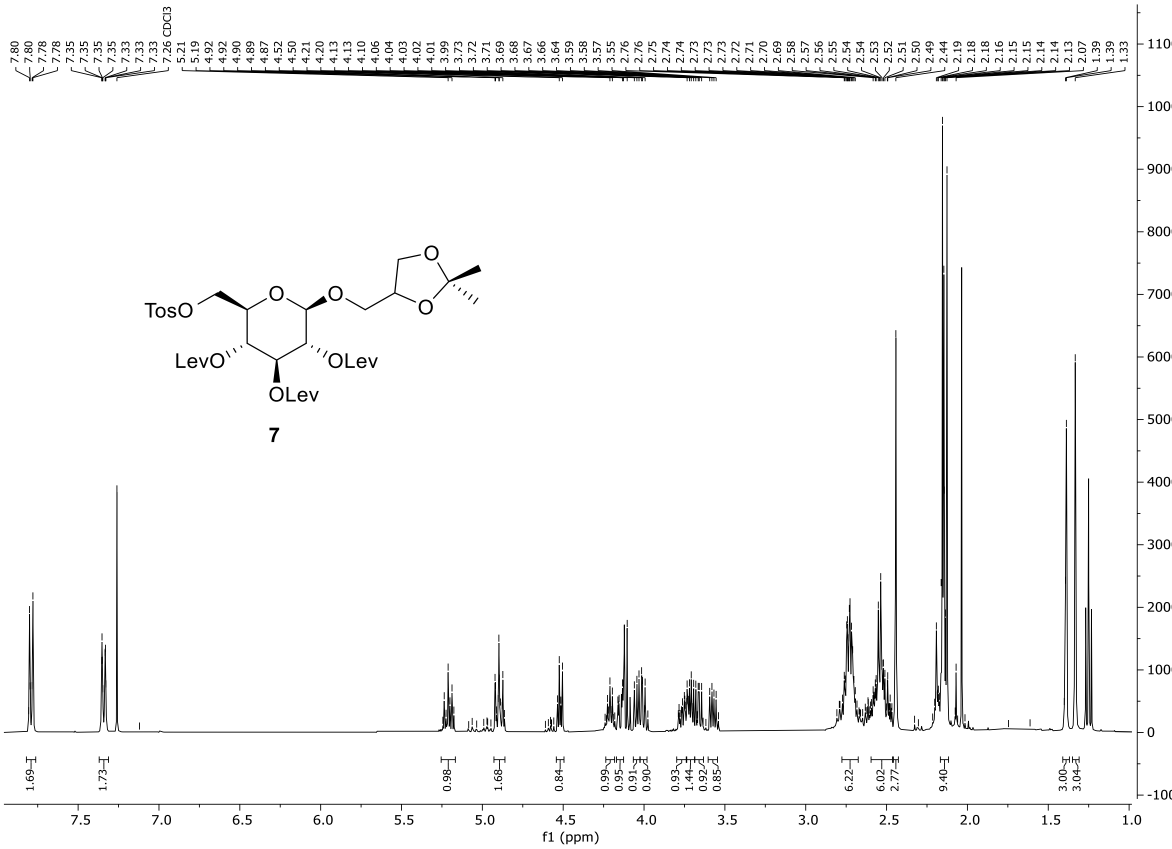

^{1}H-NMR spectrum (400 MHz, $CDCl_3$)

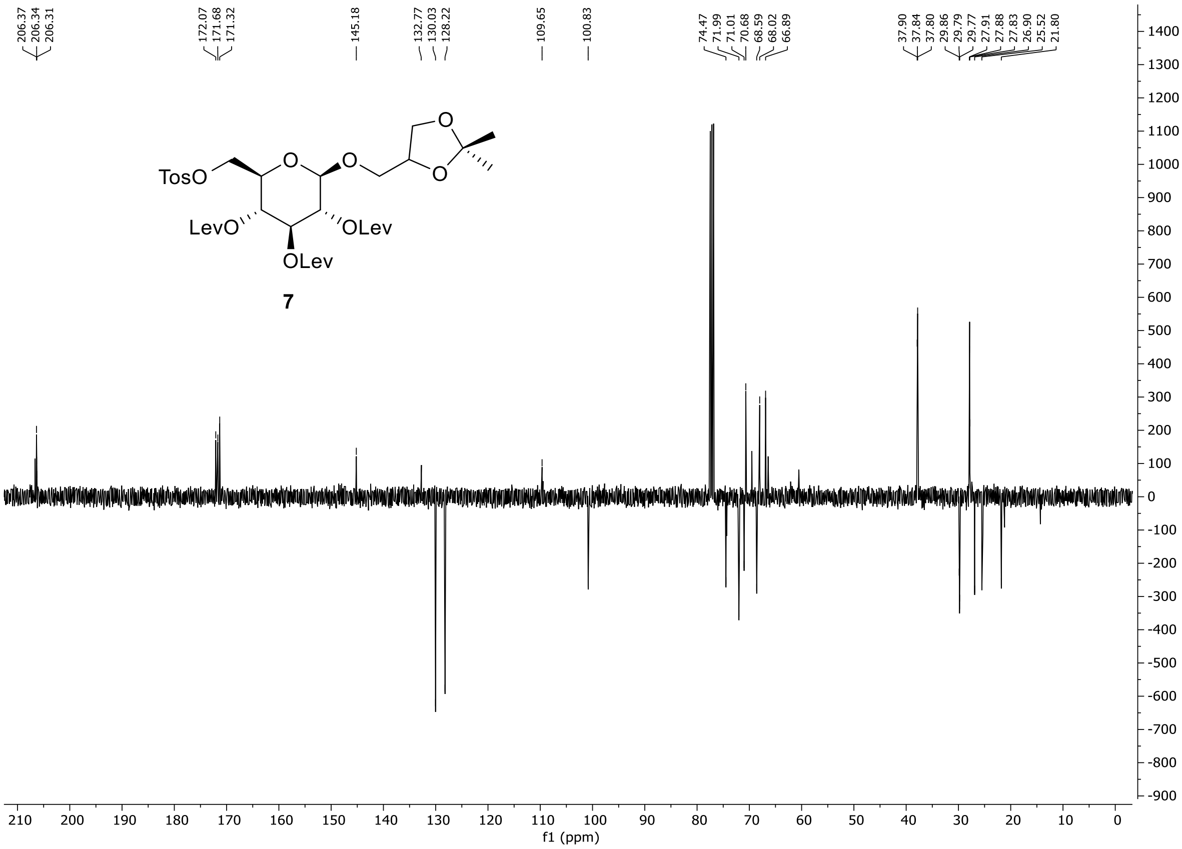

^{13}C- DEPTQ 135 NMR spectrum (100 MHz, $CDCl_3$)

1,2-Isopropylidene-3-*O*-[(2′,3′,4′-tri-*O*-levulinyl-6′-thioacetyl)-β-D-glucosyl]-(R/S)-glycerol: (8)

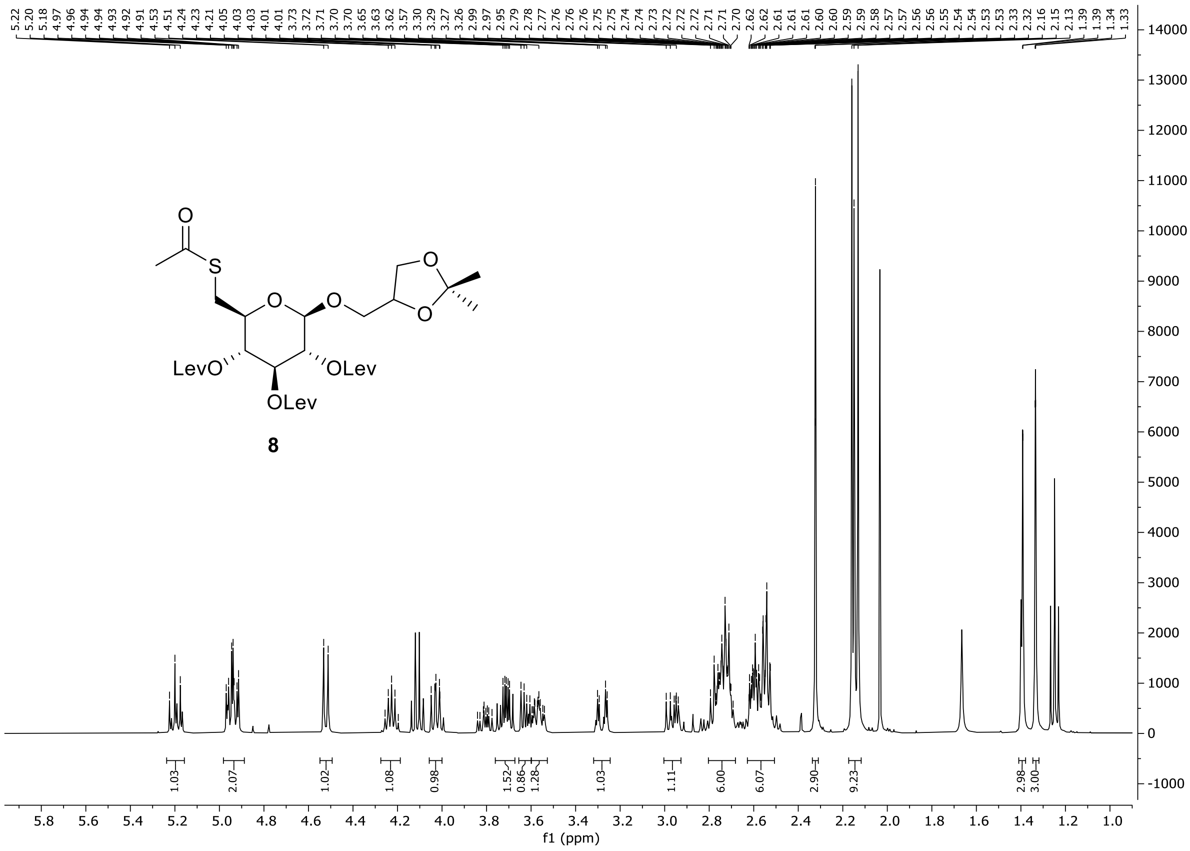

^{1}H-NMR spectrum (400 MHz, $CDCl_3$)

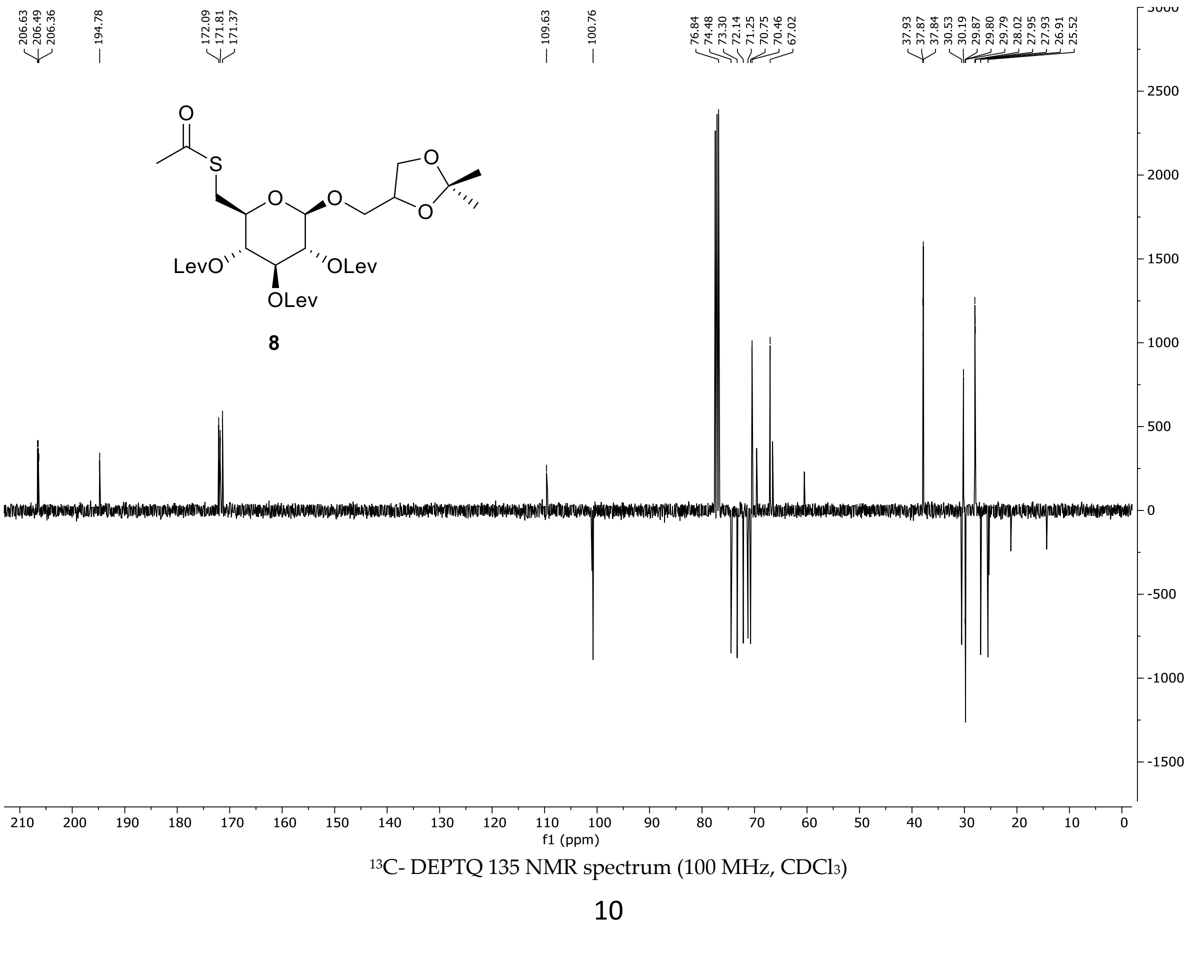

^{13}C- DEPTQ 135 NMR spectrum (100 MHz, $CDCl_3$)

3-O-[(2′,3′,4′-tri-*O*-levulinyl-6′-thioacetyl)-β-D-glucosyl]-(R/S)-glycerol (9):

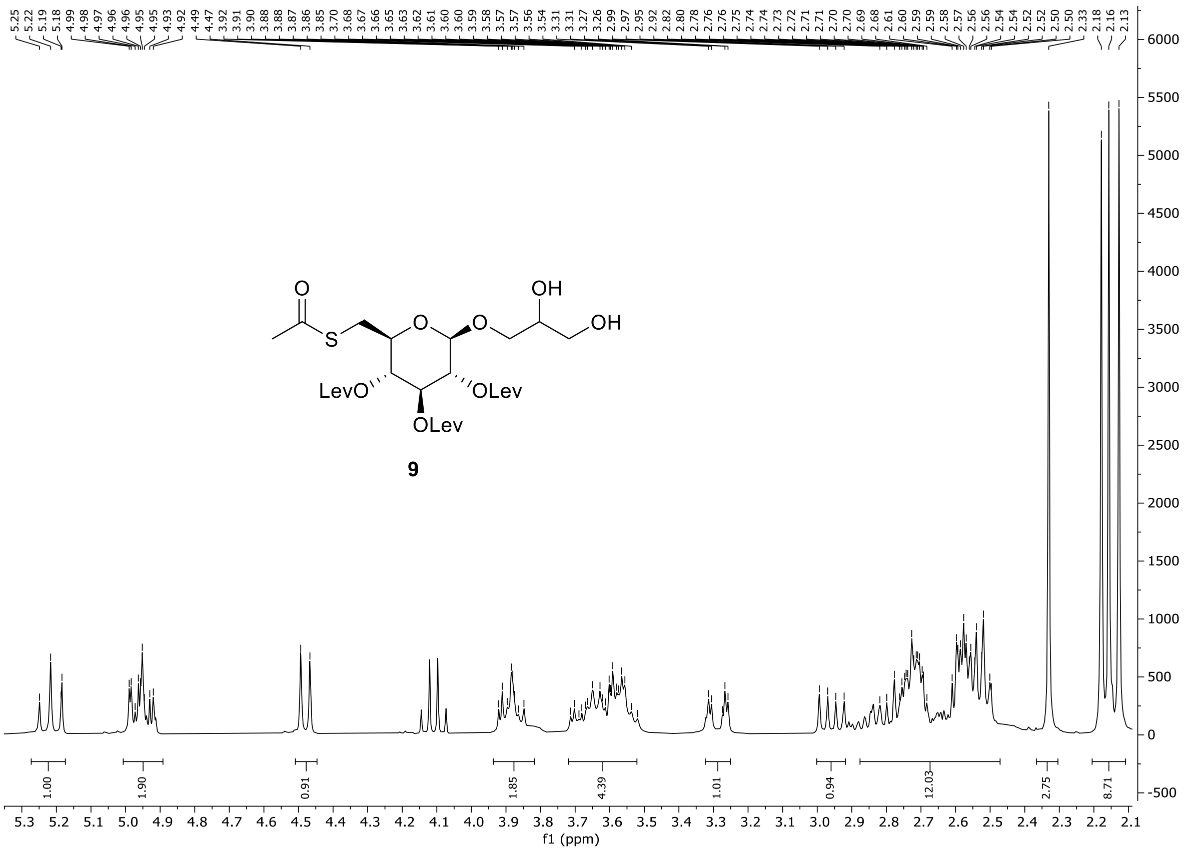

^{1}H-NMR spectrum (300 MHz, $CDCl_3$)

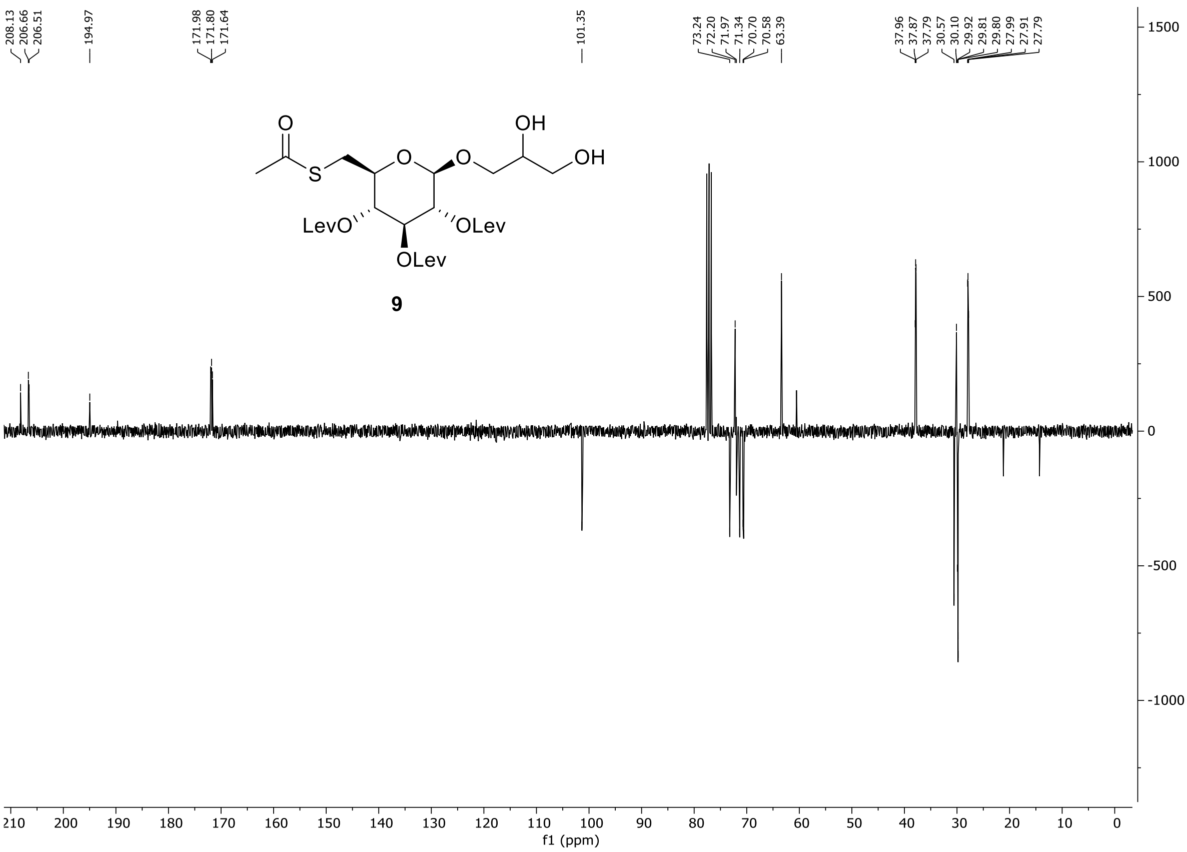

^{13}C- DEPTQ 135 NMR spectrum (75 MHz, $CDCl_3$)

1,2-distearoyl-3-*O*-[(2′,3′,4′-tri-*O*-levulinyl-6′-thioacetyl)-β-D-gluco-syl]-(R/S)-glycerol (10):

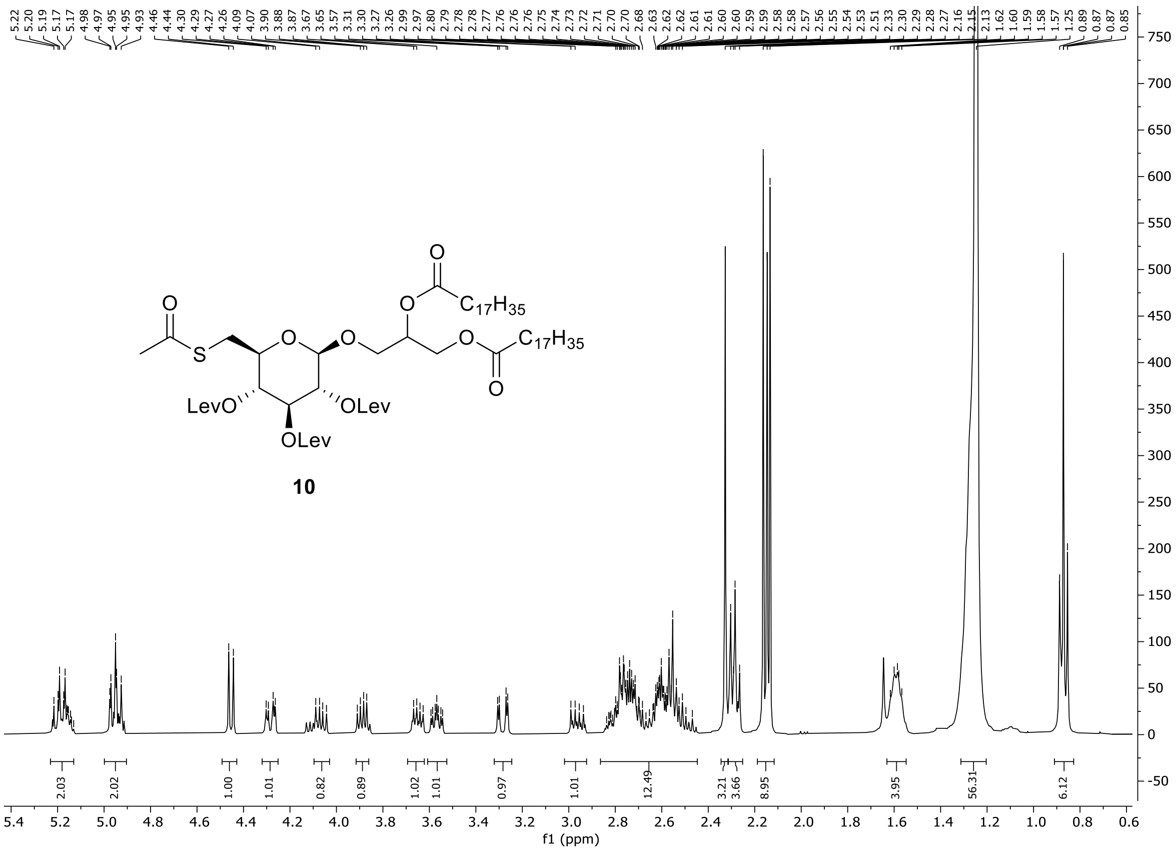

^{1}H-NMR spectrum (300 MHz, $CDCl_3$)

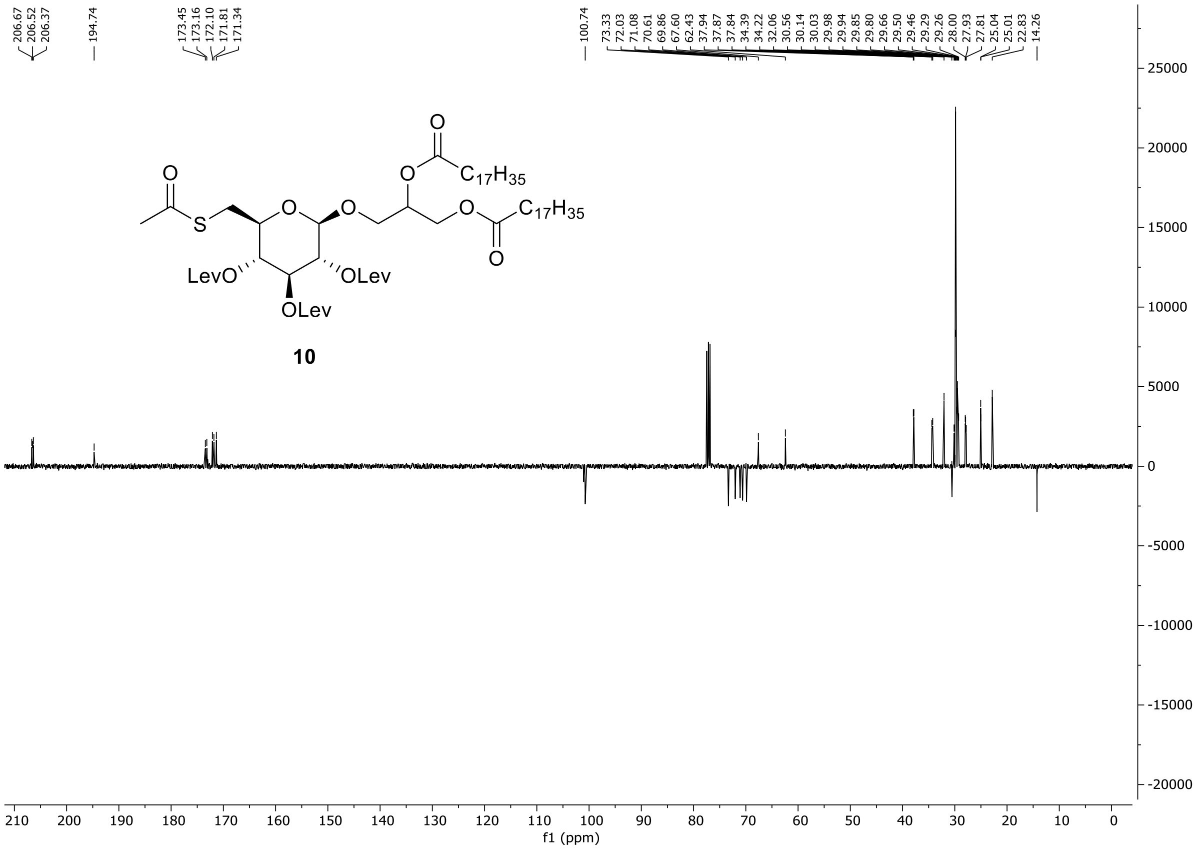

^{13}C- DEPTQ 135 NMR spectrum (75 MHz, $CDCl_3$)

1,2-distearoyl-3-*O*-[(6′-thiol)-β-D-glucosyl]-(R/S)-glycerol (11):

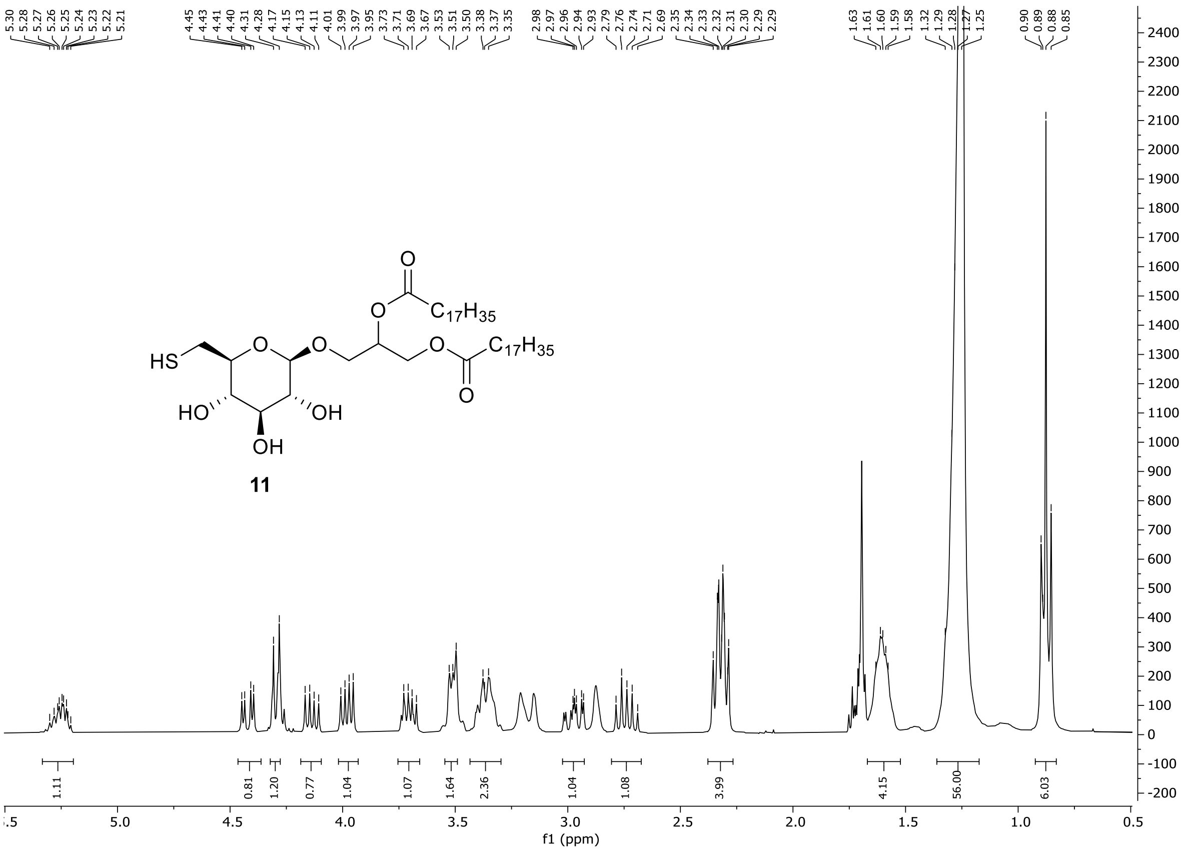

^{1}H-NMR spectrum (300 MHz, $CDCl_3$)

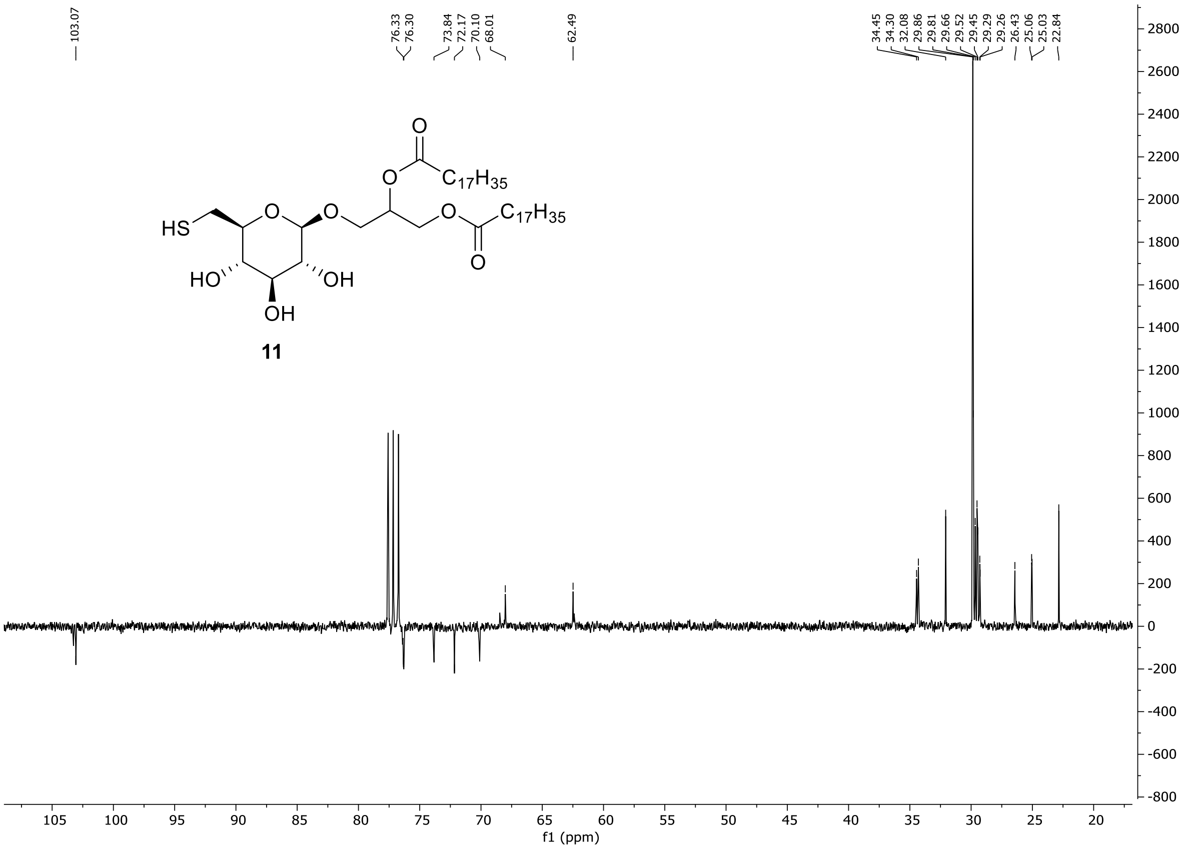

^{13}C- DEPTQ 135 NMR spectrum (75 MHz, $CDCl_3$)

1,2-distearoyl-3-*O*-[β-D-sulfoquinovosyl]-(R/S)-glycerol-potassium salt (12):

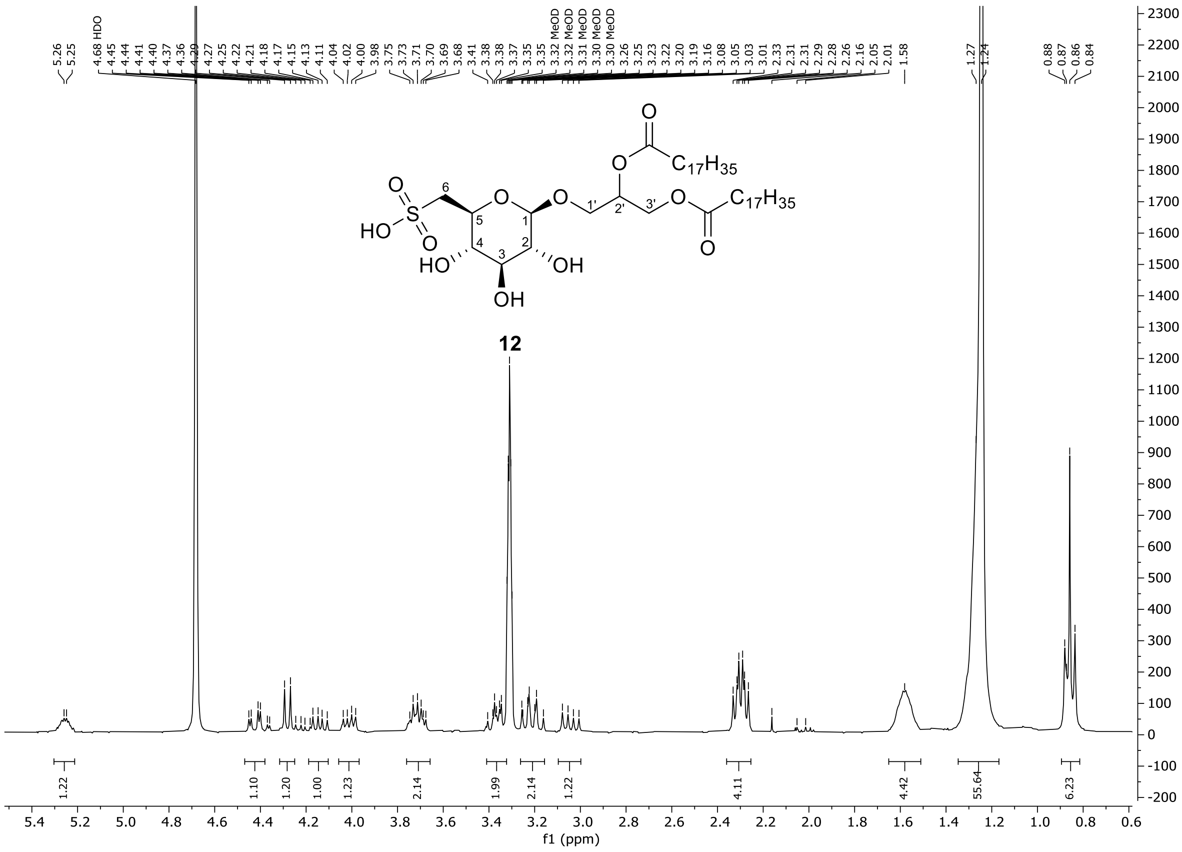

^{1}H-NMR spectrum (300 MHz, $CDCl_3$/MeOD)

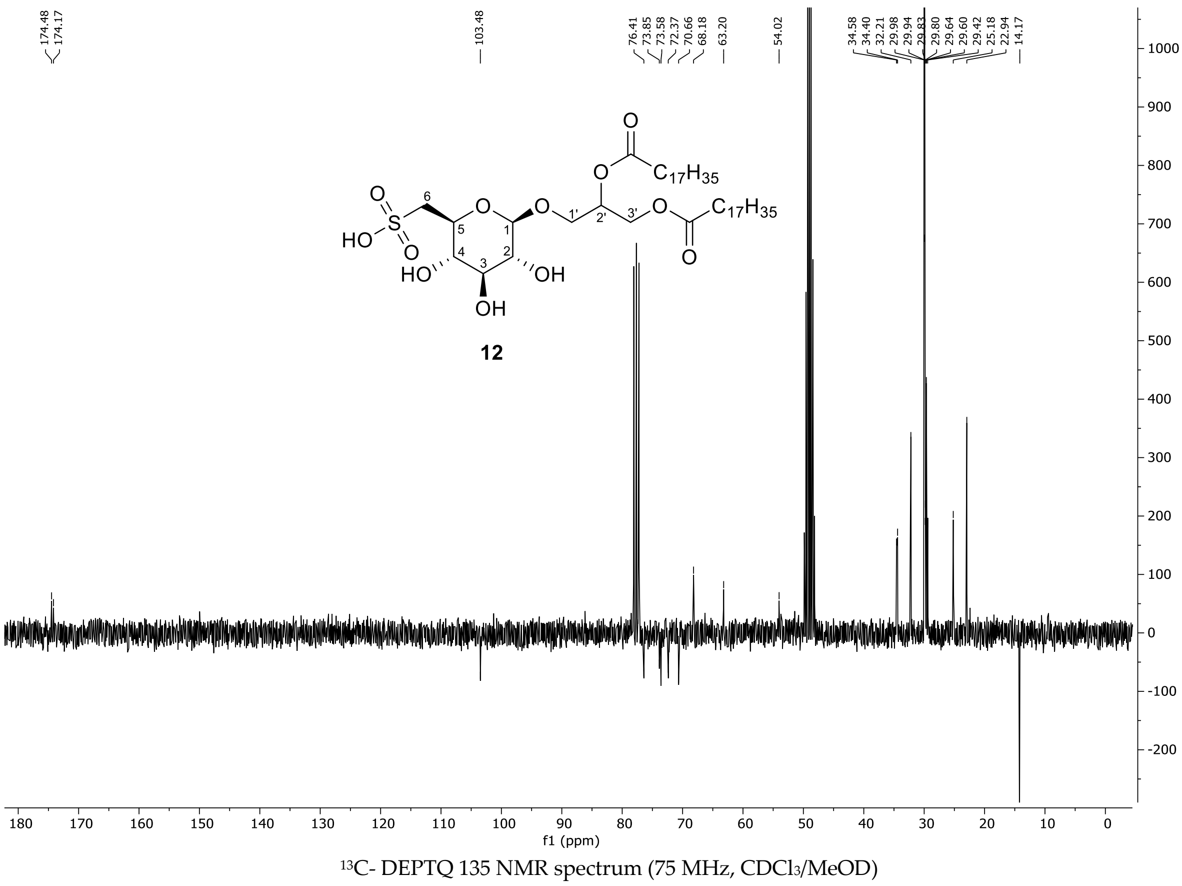

^{13}C- DEPTQ 135 NMR spectrum (75 MHz, $CDCl_3$/MeOD)
